# Micro-combinatorial sampling of the optical properties of hydrogenated amorphous $\hbox {Si}_{1-x}\,\hbox {Ge}_{{x}}$ for the entire range of compositions towards a database for optoelectronics

**DOI:** 10.1038/s41598-020-74881-5

**Published:** 2020-11-06

**Authors:** Benjamin Kalas, Zsolt Zolnai, György Sáfrán, Miklós Serényi, Emil Agocs, Tivadar Lohner, Attila Nemeth, Nguyen Quoc Khánh, Miklós Fried, Peter Petrik

**Affiliations:** 1 Institute for Technical Physics and Materials Science, Centre for Energy Research, Konkoly-Thege Rd. 29-33, 1121 Budapest, Hungary; 2 Institute for Particle and Nuclear Physics, Wigner Research Centre for Physics, Konkoly-Thege Rd. 29-33, 1121 Budapest, Hungary; 3 Institute of Microelectronics and Technology, Óbuda University, Tavaszmezo u. 17, 1084 Budapest, Hungary

**Keywords:** Surfaces, interfaces and thin films, Silicon photonics, Optical spectroscopy

## Abstract

The optical parameters of hydrogenated amorphous a-$\hbox {Si}_{1-x}\,\hbox {Ge}_{{x}}$:H layers were measured with focused beam mapping ellipsometry for photon energies from 0.7 to 6.5 eV. The applied single-sample micro-combinatorial technique enables the preparation of a-$\hbox {Si}_{1-x}\,\hbox {Ge}_{{x}}$:H with full range composition spread. Linearly variable composition profile was revealed along the 20 mm long gradient part of the sample by Rutherford backscattering spectrometry and elastic recoil detection analysis. The Cody-Lorentz approach was identified as the best method to describe the optical dispersion of the alloy. The effect of incorporated H on the optical absorption is explained by the lowering of the density of localized states in the mobility gap. It is shown that in the low-dispersion near infrared range the refractive index of the a-$\hbox {Si}_{1-x}\,\hbox {Ge}_x$ alloy can be comprehended as a linear combination of the optical parameters of the components. The micro-combinatorial sample preparation with mapping ellipsometry is not only suitable for the fabrication of samples with controlled lateral distribution of the concentrations, but also opens new prospects in creating databases of compounds for optical and optoelectonic applications.

## Introduction

Silicon germanium (SiGe) thin film research is largely inspired by assorted applications in several semiconductor devices, such as fibre optics^1^, sensors^2^, solar cells^3^, thin film transistors^4^, Schottky diodes^5^, temperature sensors^6^ and bolometers^7^. This activity is being continued with recent papers on new tenders such as mid-infrared photonic circuits (mid-IR PCs)^8^, photovoltaics^9^, supercontinuum waveguides^10^, nanowire field-effect transistors^11^, and the huge number of applications projected over the 2–20 $\upmu$m wavelength ranges^12,13^.

Until now, a great number of integrated circuit applications have been introduced, which can operate in the infrared wavelength region, e.g. at 4.5 $\upmu$m and even beyond, toward the longer wavelengths^10,14–20^. Among these applications the popularity of germanium-rich silicon-germanium (Ge-rich SiGe) has been steadily increasing, motivated mainly by its wide transparency range in the wavelength domain of importance and its strong third-order non-linearity^21^. For a waveguide application a smooth transition from Si to Ge in composition is of great significance, since a gradient in the refractive index (*n*) is essential for the optical mode confinement. Furthermore, a linearly graded SiGe layer has also the benefit that it can minimize the density of any undesirable dislocations possibly be present because of the lattice mismatch^22^. Further applications can also benefit from the careful construction of such graded layers. New opportunities in the field of modal confinement, birefringence or dispersion shaping^8^ can improve the conventionally used optical structures. These advantages of Ge-rich SiGe offer new directions in improvement for next generation spectroscopic methods operating at the infrared range, e.g. for mid-IR interferometers^23^.

The composition dependent optical properties of crystalline silicon-germanium (c-$\hbox {Si}_{1-x}\,\hbox {Ge}_{{x}}$) layers grown by chemical vapor deposition have been studied covering partly^24^ the range of compositions *x*. Optical properties of c-SiGe prepared by epitaxial growth^25^, microcrystalline layers by deposition^26^, and by ion beam amorphization^27^, with a final high-temperature crystallization process have been published for the range of $0< x < 0.3$ completely. The primary means of characterization has long been the optical method of spectroscopic ellipsometry (SE) that provides *in situ* monitoring capability during layer formation mainly in the UV-visible wavelength range^28^, (for $0< x < 0.15$), using the mid-IR in a few cases ($x = 0.1$)^29^.

One of the most important – and actually rather unique – property of a bulk SiGe system is the possibility of mixing the two components over the whole range of combinations. However, there are some inevitable hardship during the fabrication of SiGe wafers with reliable quality, since there is a large splitting of the solid/liquid phase boundary^30^. This also means that the availability of data on the optical properties of high-quality bulk SiGe (especially around $x=0.5$) is very limited.

In spite of this fact, systematic data for the amorphous compositions in the whole range of *x* have been published for amorphous silicon-germanium (a-$\hbox {Si}_{1-x}\,\hbox {Ge}_{{x}}$)^31^. The effect of all the compositions on the properties, however, has not been investigated in detail. In most of the reports relatively large deposition rates were used as a rule. Most of these publications introduce a low Ge concentration and only these films have provided properties that are compatible with opto-electronic devices. The structural and electrical properties of a-SiGe alloys have also been investigated^32^, including a broader range of compositions for a-$\hbox {Si}_{1-x}\,\hbox {Ge}_{{x}}$ films using $x = 0.0$, 0.1, 0.2, 0.5 and 1.0^33^.

The biggest commercial use of SiGe alloys concentrated on hydrogenated a-SiGe (a-$\hbox {Si}_{1-x}\,\hbox {Ge}_{{x}}$:H). It has been studied extensively because of its potential for use in thin film solar cells^34^. It is well known that the a-$\hbox {Si}_{1-x}\,\hbox {Ge}_{{x}}$:H alloys are used in multi-junction solar cells in order to increase the efficiency of the cells^15,16,35,36^. One of the advantages of this alloy material is the possibility to vary the band gap with the Ge concentration^25,37^ and thus to optimize the efficiency of the solar spectrum. The plasma enhanced chemical vapour deposition (PECVD) technique is known for producing amorphous thin layers with atomic proportions changing with the composition of the gas feed and containing a few percentage of H: thus the deposited layer contains Si-H and Ge-H covalent bonds with a bond strength between 3.3 and 3.0 eV, respectively. The advantage of this technology is that the defect density decreases from $1\times 10^{20}$ to $5\times 10^{15}$ $\hbox {cm}^{-3}$. The a-$\hbox {Si}_{1-x}\,\hbox {Ge}_{{x}}$:H thin films can be characterized by the variations of the composition and the optical and electrical properties using the results of Refs.^32,37^. These variations are not linear and it is necessary to take into account the increase of H levels when the composition goes to Si-rich alloy, because the H preferentially bonds to Si, i.e. the H content decreases for increasing *x*^17^. In spite of this fact, the refractive index (*n*) of the SiGe alloy was estimated by Brun et al.^14^ using the linear formula of $n_{Si_{1-x}Ge_{x}} = x\times n_{Ge}+(1-x)\times n_{Si}$, where $n_{Si}$ and $n_{Ge}$ were taken from Ref.^18^. This formula was named Vegard’s law-like formula by the authors of Ref.^38^.

This work focuses on the identification of wavelength and photon energy ranges for the Vegard’s law-like behavior of *n* for a-$\hbox {Si}_{1-x}\,\hbox {Ge}_{{x}}$:H for wavelengths ranging from 190 to 1690 nm (photon energies from 0.7 to 6.5 eV) in the whole range of the composition from $x = 0$ to $x = 1$. The potential impact of our work is shown by the fact that Si-based technologies became significant for new mid-IR PCs recently, following a trend of telecom wavelength devices^14^. SiGe-on-Si is particularly interesting since it allows the control of properties such as *n* or the band gap ($E_g$) by controling the Ge concentration, while extending the range of operation up to at least 14 $\upmu$m^8,14^. In this article we show that not only the strictly controlled preparation of a-$\hbox {Si}_{1-x}\,\hbox {Ge}_{{x}}$:H films is possible over the entire range of $0 \le x \le 1$ using magnetron sputtering over a length of 2 cm, but—for wavelength ranges we identify in the study—the composition, and even more importantly the optical gap ($E_g$) and *n* all show an accurately linear dependence on the position.

## Results

### Preparation of a-$\hbox {Si}_{1-x}\,\hbox {Ge}_{{x}}$:H using “single-sample” micro-combinatory

**Figure 1:**
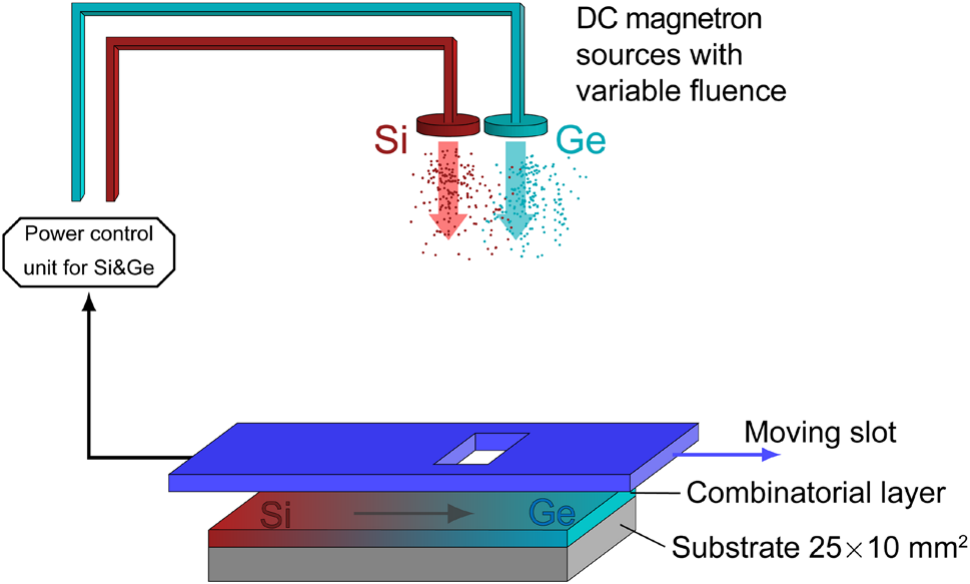
Setup used for the “single-sample concept” combinatorial deposition of the a-$\hbox {Si}_{1-x}\,\hbox {Ge}_{{x}}$:H layers.

a-$\hbox {Si}_{1-x}\,\hbox {Ge}_{{x}}$:H samples were prepared on 10 mm $\times$ 25 mm size Si wafers by “single-sample” micro-combinatory that resulted in gradient composition of a-$\hbox {Si}_{1-x}\,\hbox {Ge}_{{x}}$ with *x* ranging in $0 \le x \le 1$. The layers with thicknesses of about 100 nm were deposited in a stainless steel UHV system by dual DC magnetron sputtering using a scaled-up device^19^ originally developed for synthesizing micro-combinatorial transmission electron microscopy samples. The present arrangement sweeps a shutter with a 1 mm $\times$ 10 mm slot in fine steps above the wafer meanwhile the power of the two magnetron sources is regulated in sync with the slot movement. As the slot passes over the substrate, the fluence of Si gradually decreases from 100 to 0%, while that of Ge increases from 0 to 100%, that creates the required gradient of the composition. The details of the sample preparation were described earlier for non-hydrogenated a-$\hbox {Si}_{1-x}\,\hbox {Ge}_{{x}}$ samples^31^. In this work, the hydrogenated a-$\hbox {Si}_{1-x}\,\hbox {Ge}_{{x}}$:H layers were deposited at a sputtering rate of 0.4 nm/s. The distance of the substrate was 12 cm from the targets of 2-inch diameter. The DC magnetron sputtering was performed with a mixture of high-purity H and Ar gases that were introduced into the chamber via separate flanges placed at equal distances of about 20 cm and 35 cm from the two targets, respectively. The H flow rates were kept constant to achieve the desired partial pressure value (*p*$_H$) and the total plasma pressure (*p*) was kept at $3 \times 10\,^{-3}$ mbar by the regulation of the additional Ar gas inflow. The scheme in Fig. 1 depicts the experimental arrangement and the construction of the combinatorial specimen. The 25 mm long substrates exhibit a 20 mm long gradient $\hbox {Si}_{1-x}\,\hbox {Ge}_{{x}}$ track enclosed between 2.5 mm long lead-in sections of one target’s flux. The sample position “*0*” belongs to the Si-rich side of the gradient track.

### Determination of the H content using elastic recoil detection analysis in the presence of H loss during measurement

**Figure 2:**
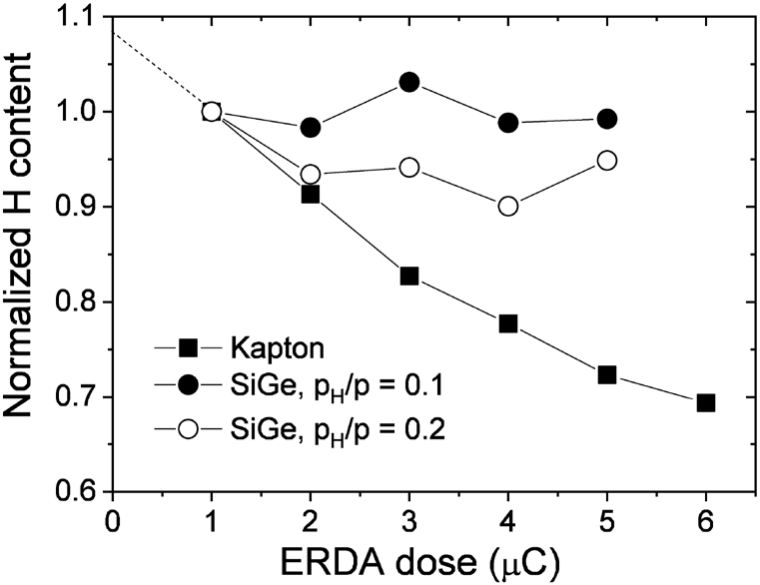
Normalized H contents as a function of the measurement dose for 1.6 MeV $\hbox {He}^+$ ERDA experiments. Squares represent data for Kapton as reference for H, while full and open dots denote samples ‘C’ and ‘D’ ($p_H/p = 0.1$ and 0.2), respectively. The dotted line shows linear extrapolation to zero dose for sample ‘D’.

Figure 2 shows normalized H contents, i.e., normalized H peak integrals in the elastic recoil detection analysis (ERDA) spectra, as a function of the measurement dose for 1.6 MeV $\hbox {He}^+$ ERDA experiments. Data for both Kapton (C22H10N2O5) and for a-$\hbox {Si}_{1-x}\,\hbox {Ge}_{{x}}$:H layers with $p_H/p = 0.1$ and 0.2 (samples ‘C’ and ‘D’, respectively) are shown. Kapton is a reference calibration sample with well known high H content and it is used to quantitatively determine the amount of H in the a-$\hbox {Si}_{1-x}\,\hbox {Ge}_{{x}}$:H layers. As Fig. 2 shows, by increasing the ERDA measurement dose, significant loss of H occurs especially in Kapton, but also in the a-$\hbox {Si}_{1-x}\,\hbox {Ge}_{{x}}$:H layer of $p_H/p = 0.2$ (sample ‘D’ in Table 1). For sample ‘C’ ($p_H/p = 0.1$), however, no significant change in the amount of H can be estimated within the applied dose range. The dotted line shows extrapolation of the amount of H to zero measurement dose for sample ‘D’ ($p_H/p = 0.2$). In this case a H loss of about 17% can be estimated for a measurement dose of 2 $\upmu$C. The same dose of 2 $\upmu$C was applied in the Rutherford backscattering spectrometry (RBS) and ERDA measurements performed in this work. After the ERDA analysis, the H content was evaluated from the measured ERDA spectra, taking into account the result of H loss experiments for a dose of 2 $\upmu$C.

**Table 1:** Parameters of sample preparations. $p_H/p$ lists the ratio of the applied partial pressure ($p_H$) to the total plasma pressure ($p=3\times 10^{-3}$ mbar), $P_{0,Si}$ and $P_{0,Ge}$ are the maximum sputtering power.

Sample	$p_H/p$	$P_{Si}^0$ (W)	$P_{Ge}^0$ (W)	*d* (Si,Ge) (nm)	*n* (Si,Ge) @ 633 nm
A	0.00	310	86	($101.58 \pm 0.13,$ $106.87 \pm 0.15$)	(4.69, 5.11)
B	0.05	310	86	($124.17 \pm 0.11,$ $141.10 \pm 0.15$)	(4.70, 5.07)
C	0.10	310	86	($104.75 \pm 0.16,$ $104.52 \pm 0.13$)	(4.34, 4.99)
D	0.20	310	86	($100.01 \pm 0.07,$ $101.50 \pm 0.15$)	(4.16, 5.01)

*d* and *n* denote the layer thickness and the refractive index at the wavelength of 633 nm on the Si-rich and Ge-rich sides of the wafer calculated using the best optical model described in section “Parameterization of the dielectric function”. The sputtering rate was 0.4 nm/s in each case.

### Linear dependence of composition on the lateral position revealed by RBS and ERDA

**Figure 3:**
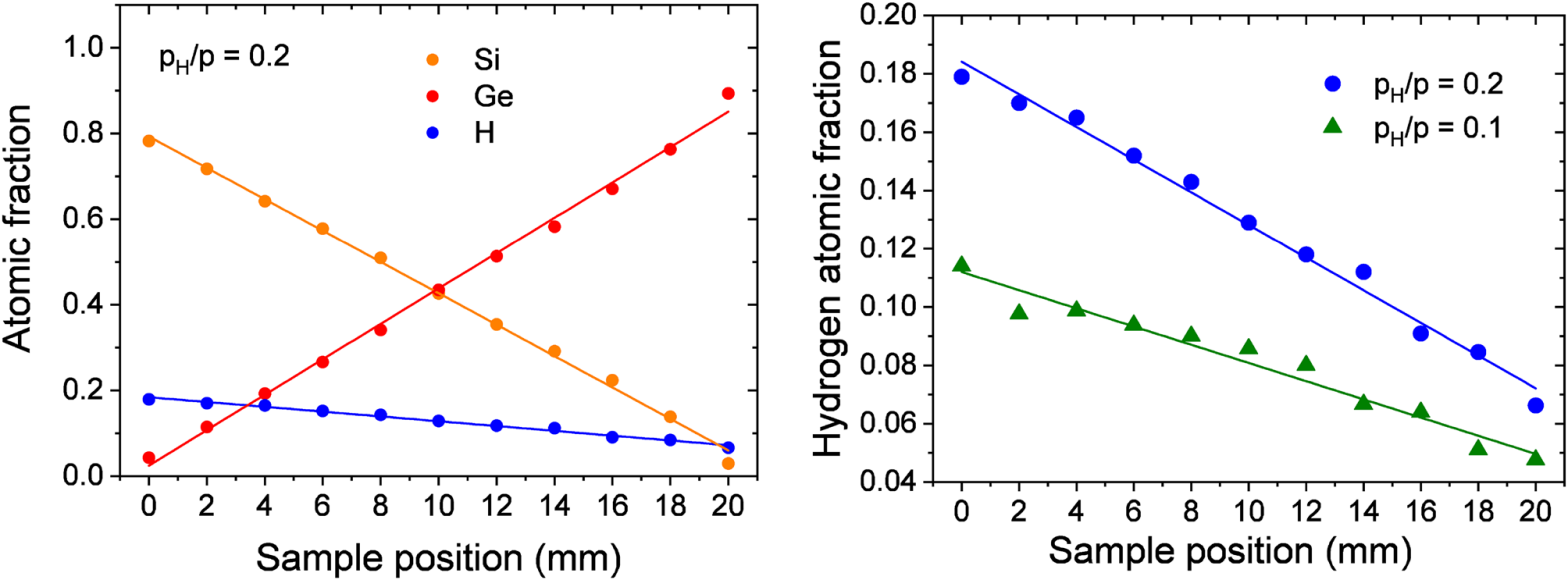
Left-hand side: atomic fractions of Si and Ge showing the incorporated H for $p_H/p=0.2$ of the SiGe layer along the 20 mm length of the sample measured by RBS. Right-hand side: atomic fractions of H evaluated from ERDA spectra applying spectrum simulations by the RBX software^39^ for samples ‘C’ and ‘D’ ($p_H/p = 0.1$ and 0.2, respectively). Solid lines show linear fits to the data. Note that the test results of H loss were taken into account in the evaluation of the H content as shown in Fig. 2.

Atomic fractions of Si, Ge and H for sample ‘D’ ($p_H/p = 0.2$) are shown in Fig. 3 as functions of the lateral position along the center line of the sample parallel to its long edge. The Si and Ge contents were evaluated from 1.6 MeV $\hbox {He}^+$ RBS spectra and the H contents from 1.6 MeV $\hbox {He}^+$ ERDA spectra, using the RBX software^39^. For further details of the RBS/ERDA experiments see section “Methods4”. Note that in Fig. 3 the results of H loss experiments were also taken into account in the evaluation of the H contents as described in the previous section. The solid lines show linear fits to the experimental data. A linear dependence of the Si, Ge, and H contents vs. the sample position can clearly be assigned. The Si atomic fraction drops from 0.8 (zero sample position) to about zero (sample position of 20 mm), meanwhile the H atomic fraction drops from about 0.2 to less than 0.1, respectively. Therefore, a correlation between the H content and the Si:Ge ratio can be stated in the full range of a-$\hbox {Si}_{1-x}\,\hbox {Ge}_{{x}}$ compositions. We emphasize that similar trends were found also for sample ‘C’ ($p_H/p = 0.1$), see the next section for the H content.

### Incorporation of H

A summary of the H content depending on the preparation conditions is plotted in Fig. 3. The solid lines show linear fits to the experimental data. Note that the results of test measurements for the H loss were taken into account in the evaluation of the H contents. A linear dependence of the H content vs. the sample position can be assigned for each sample. The higher the Si/Ge ratio, the higher the H content, in agreement with previous investigation up to a Ge concentration of $x=0.6$^40^. We have also found that the lateral distribution of the Si/Ge ratio is not influenced by $p_H/p$. Furthermore, the slope for sample ‘D’ ($p_H/p = 0.2$, blue curve) significantly differs from that of sample ‘C’ with a smaller H content, because a higher partial pressure of H was applied during the sputtering process. The highest H incorporation is close to the nominal H gas content value at the Si-rich edge of the sample (sample position of zero). At the Ge-rich edge of the sample (position of 20 mm), however, the amount of incorporated H is dropped by a factor of 2.2 for sample ‘C’ ($p_H/p = 0.1$), and by a factor of 2.5 for sample ‘D’ ($p_H/p = 0.2$), as compared to the Si-rich edge of the sample. The theoretical background of the concentration dependence of H incorporation will be given in section “Discussion” below.

### Optical properties

The features of the micro-combinatorial sample enable the determination of the optical properties with high spatial and composition resolutions. The width of the focused light spot is $\approx$0.3 mm that, considering a 20 mm long full range gradient section, corresponds to a resolution of $\approx$ 0.015 in *x*, which is equivalent with 1.5 at%. In this work we used a step size corresponding to the size of the focused spot. However, the resolution may be increased even further when using step sizes smaller than the spot and applying a method for lateral inhomogeneity^41^. The equipment used for the measurement allows a lateral step size far below the spot size.

**Figure 4:**
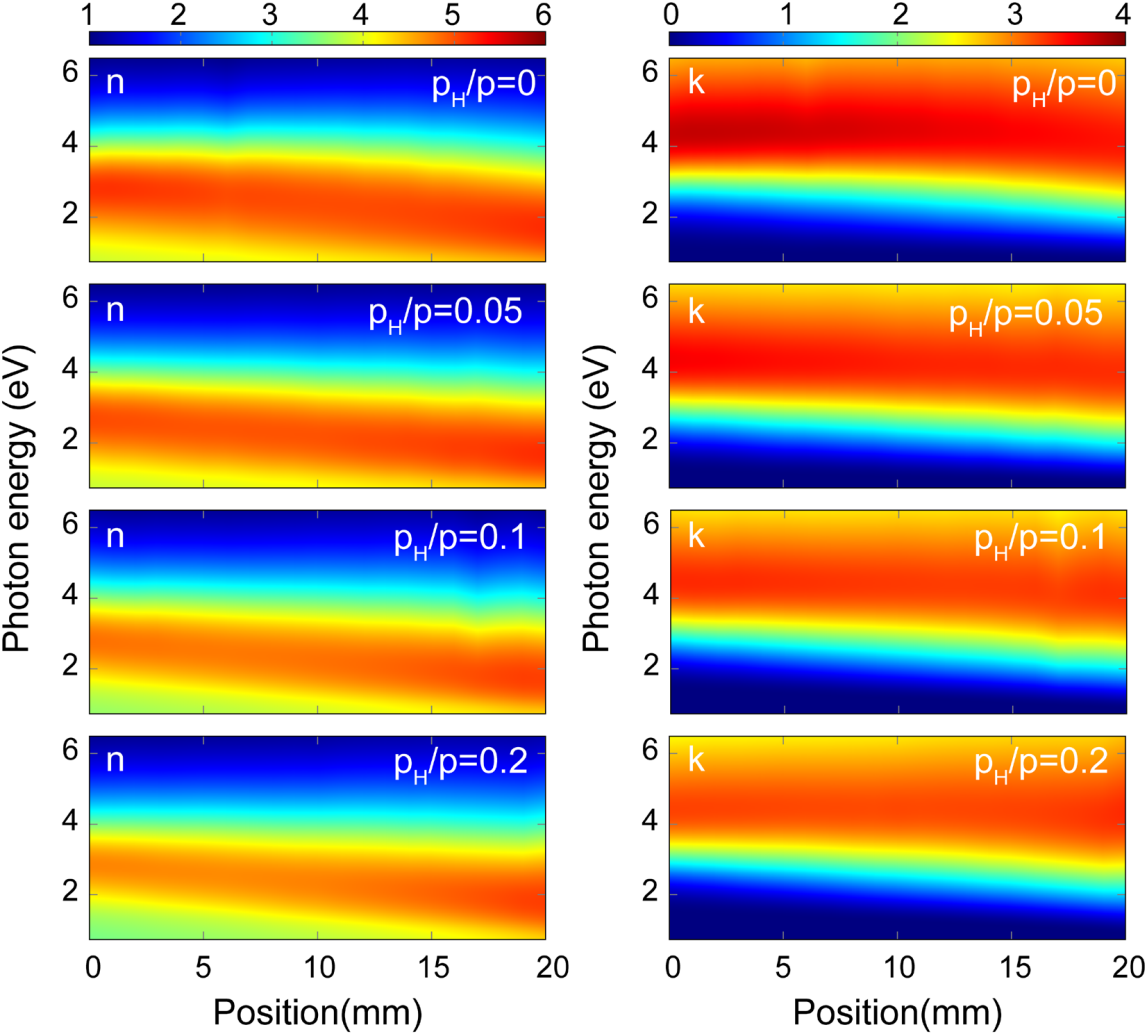
Real and imaginary parts of the complex refractive indices of a-$\hbox {Si}_{1-x}\,\hbox {Ge}_x$:H thin films (left and right column, respectively) with different partial pressures of H ($p_H/p = 0, 0.05$, 0.1 and 0.2) as a function of both the lateral position along the 20 mm long gradient section and photon energy. In accordance with the RBS plots, the zero position corresponds to the Si-rich side of the sample. (A Supplementary Information S1 with all the values plotted here are attached to the article.) (Spectra of $p_H/p = 0.1$ is removed at position 8 mm).

**Figure 5:**
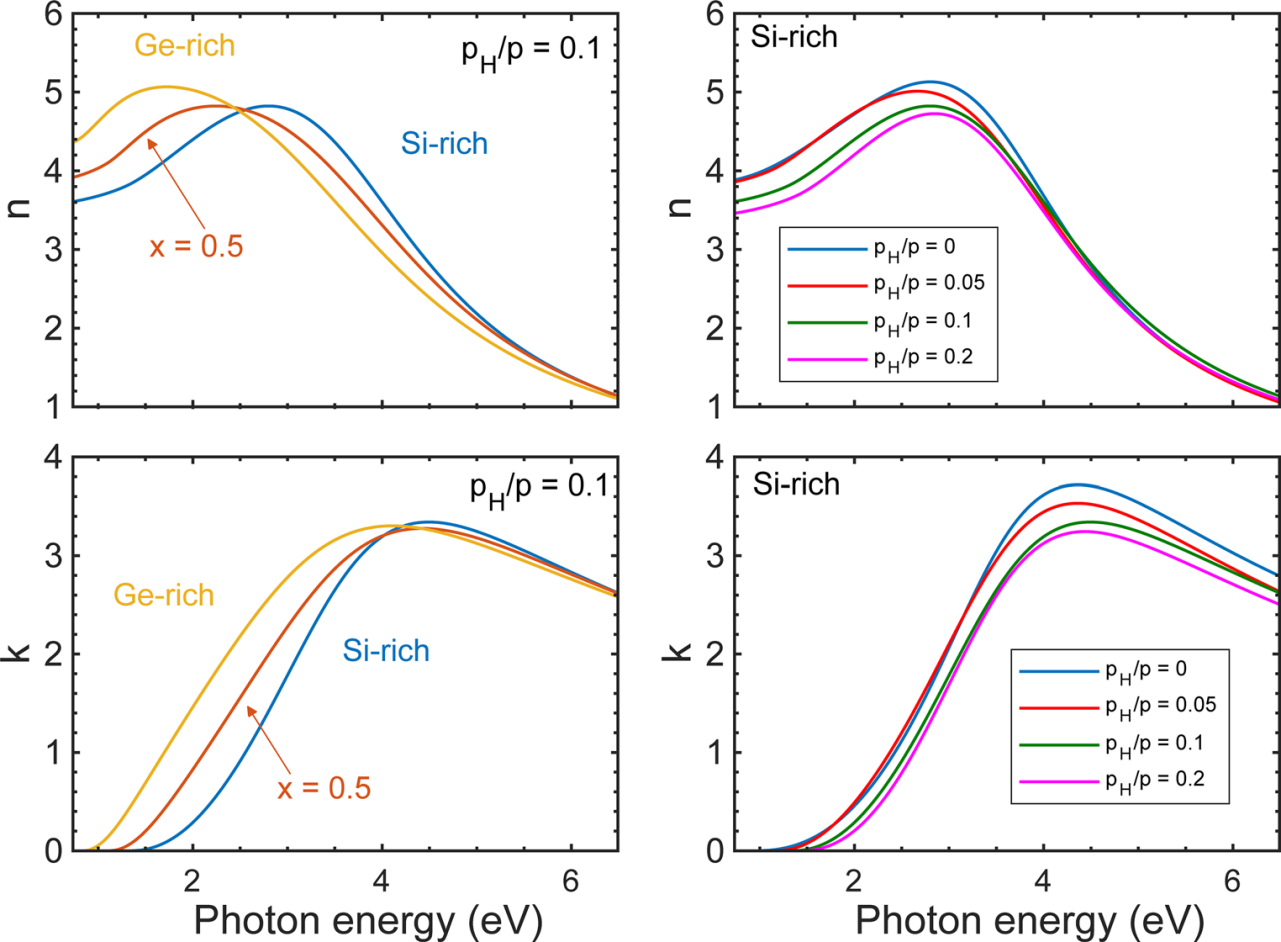
Real and imaginary parts of the complex refractive indices of a-$\hbox {Si}_{1-x}\,\hbox {Ge}_{{x}}$:H with $p_H/p=0.1$ for different compositions (left-hand side) and for different H contents for the Si-rich side given as $p_H/p$ values written next to the corresponding curves (right-hand side).

Figure 4 shows maps of both *n* and the extinction coefficient (*k*) of all the samples ($p_H/p = 0.0$, 0.05, 0.1 and 0.2) in the whole range of compositions and for photon energies from 0.7 to 6.5 eV. *n* and *k* were obtained by fitting the measured spectra using the Cody–Lorentz (CL) optical model described in sections of “Discussion” and “Methods”. The features in both *n* and *k* show a linear change with the position and hence also with the composition. The single broad peak, characteristic of amorphous semiconductors can clearly be identified in all maps. The peaks are shifted to smaller photon energies as the composition changes from Si to Ge (positions from 0 to 20 mm), as shown by Fig. 5. There is also a remarkable shift of the peaks as a function of the H concentration towards a higher band gap, smaller amplitude and broadening. A more detailed quantitative analysis in terms of the fitted oscillator parameters are given in the next section.

Although the spectra in Fig. 4 change rather smoothly with the composition, which might be evaluated by a series of samples without the combinatorial method, the benefit of the combinatorial method is three-fold. (1) The sample is prepared in one step assuring that all the parameters of the sample preparation and the substrate is the same except for the modulated parameter (the composition). (2) The measurement and the evaluation is also easier and quicker, because it can automatically be performed by a lateral scan over the sample surface, and the evaluation process also supports a single-process interpretation. (3) Although the maps in Fig. 4 reveal smooth changes, it will be shown below that the variation of the optical properties is not linear in all the photon energy ranges. The combinatorial method also allows the characterization of larger modulations and unexpected variations of the properties without any assumption of the lateral dependence.

## Discussion

### Parameterization of the dielectric function

Optical properties of a-$\hbox {Si}_{1-x}\,\hbox {Ge}_{{x}}$:H were studied using SE (to analyze the spectral behavior of the dielectric function) and absorption spectroscopy (for the determination of the band gap energy). The Tauc or Tauc–Lorentz (TL) methods were applied for the determination of the band gap energy using absorption spectroscopy in transmission mode^42^. In order to characterize the above-gap features and *n* values, SE was used in reflection mode in order to analyze second derivatives^25^ or model dielectric functions^43^ to characterize the critical point features (mainly in crystalline materials and for a limited range of compositions). In the above-gap wavelength range, the main features of the optical properties can usually directly be obtained from the pseudo dielectric function (the bulk equivalent response of the sample), due to the small penetration depth of light. The analysis of the pseudo dielectric function is sufficient for the determination of the critical point features^25^ in case of crystalline materials. For amorphous semiconductors, the TL parameterization^44,45^ was widely used that provides gap energies, as well as peak energy position, amplitude and broadening of the usually broad absorption features of these materials. In this approach, the imaginary part of the dielectric function, $\varepsilon _2$, which is proportional to the joint density of electron states, is described using the following formula:

1 $$\begin{aligned} \varepsilon _{2}(E) = {\left\{ \begin{array}{ll} \frac{A\cdot E_{0}\cdot \Gamma \cdot (E-E_g)^2 }{(E^2-E_g)^2+\Gamma ^2\cdot E^2}\cdot \frac{1}{E} &{} \text {if } E > E_g, \\ 0 &{} \text {if } E \le E_g, \end{array}\right. } \end{aligned}$$

where the parameters *A*, $E_0$, $E_g$ and $\Gamma$ are the amplitude, the peak in the joint density of states, the gap energy and the broadening parameter, respectively, all of them in unit of eV. The real part of the dielectric function $\varepsilon _1$ is obtained by performing a Kramers–Kronig integration of $\varepsilon _{2}$:

2 $$\begin{aligned} \varepsilon _{1}=\varepsilon _{1}(\infty )+\frac{2}{\pi }P\int _{E_{g}}^\infty \frac{\xi \varepsilon _{2}(E)}{\xi ^2-E^2}\text {d}\xi , \end{aligned}$$

where *P* stands for the Cauchy principal part of the integral and an additional parameter $\varepsilon _{1}(\infty )$ was included. Normally, $\varepsilon _{1}(\infty ) = 1$. Besides the fact that it applies only a small number of fit parameters, one of the most important features of the TL approach published by Jellison et al.^45^ is that $\varepsilon _1$ can be given by an analytical formula that can be calculated at a high speed, avoiding the time-consuming numerical integration using Eq. (2). The complex dielectric function is calculated then by the equation of $\varepsilon = \varepsilon _{1}+i\varepsilon _{2}$, and $\varepsilon = (n+ik)^2$.

The TL dispersion term is a successful model for describing amorphous materials^46^, however, it has some limitations as well, such as the fact that the shape of the interband absorption onset for a-Si is closely consistent with a formula derived on the assumption of parabolic bands and a constant dipole matrix element^47,48^, i.e. $\varepsilon _2(E)\propto (E-E_g)^2$. The Tauc law formula, however, was derived on the assumption of parabolic bands and a constant momentum matrix element^49^, i.e. $\varepsilon _2(E)\propto [(E-E_g)^2/E^2]$. (For a comparison of both approaches see Ref.^50^).

Ferlauto et al.^48^ carried out modifications and derived the CL model of

3 $$\begin{aligned} \varepsilon _{2}(E) = {\left\{ \begin{array}{ll} \frac{E_1}{E}\exp \left[ \frac{(E-E_t)}{E_\mu } \right] &{} \text {if } 0 < E \le E_t, \\ G(E)L(E)=G(E)\frac{AE_0\Gamma E}{[(E^2-E_0^2)^2+\Gamma ^2E^2]}, &{} \text {if } E > E_t, \end{array}\right. } \end{aligned}$$

where $E_t$ is a transition energy between the Urbach tail and the band-to-band transitions, $E_\mu$ represents the extent of broadening and $E_1=E_{t}L(E_t)G(E_t)$. Here, *L*(*E*) is the Lorentz oscillator function and *G*(*E*) is a variable band edge function, a result of the constant dipole approach

4 $$\begin{aligned} G(E)=\frac{(E-E_g)^2}{(E-E_g)^2+E_p^2}, \end{aligned}$$

where $E_p$ is the transition energy that separates the absorption onset behavior from the Lorentzian behavior. Note that the Urbach term can also be used for the TL approach of Eq. 1. In this study all the parameters were fitted from the CL model (Eq. 3), except parameters $E_t$ and $E_\mu$, since these parameters turned out to be insensitive, causing huge uncertainties. These parameters were fixed during the optical analysis at values of $E_\mu =0.5$ and $E_t=0$.

Thus all together 6 parameters were fitted to describe the optical properties (*n*, *k*), including the remaining CL parameters, and two further parameters for the thickness of the a-SiGe and surface layers. The surface layer is a combination of an oxide and an $\text {\AA }$-scale roughness – see the last section of this article about the atomic force microscopy (AFM) results. From the numerous approaches investigated to model the surface overlayer (including effective medium and literature references^51,52^), the best model applied a Cauchy layer $(n(\lambda)=A+B/\lambda^2+C/\lambda^4)$ with dispersion parameters of *A* = 1.451, *B* = 0.004 and *C* = 0 determined from an oxidized Si reference wafer.

The 9th parameter was used for describing the small amount of vertical inhomogeneity. A slightly graded refractive index that was more pronounced towards the Ge-rich side of the sample. Although the grading was small (a few %), the fit quality was significantly improved by applying this parameter, which is consistent with the decrease of the root mean square error (RMSE) shown in Fig. 8. A linear optical variation (vertical inhomogeneity parameter) through the depth of the film was found (using the average value throughout the article) with reasonably small values (Fig. 8).

The parameters were obtained using a random global search in ranges of reasonable parameter values, followed by a Levenberg–Marquardt regression algorithm to fit the final value and to obtain the mathematical uncertainties, as shown in Table 2. Besides the careful adjustments of the parameter limits, uniqueness fits were also evaluated to avoid cross-correlations and local minima in the parameter space. A typical set of parameters is presented in the Table 2 for sample ‘A’.

**Table 2:** Typical parameter values obtained using CL parameterization with confidence limits of 90% for sample ‘A’.

Measurment place parameter	Si-rich end	$x=0.5$	Ge-rich end
$\varepsilon _1(\infty )$	0.44 ± 0.01	0.41 ± 0.01	0.30 ± 0.01
A (eV)	115.82 ± 0.74	93.75 ± 0.37	101.78 ± 0.34
$\Gamma$ (eV)	2.701 ± 0.003	3.355 ± 0.006	4.012 ± 0.007
$\hbox {E}_0$ (eV)	3.680 ± 0.003	3.896 ± 0.003	3.561 ± 0.004
$\hbox {E}_g$ (eV)	0.949 ± 0.003	0.847 ± 0.004	0.692 ± 0.004
$\hbox {E}_p$ (eV)	1.95 ± 0.02	0.95 ± 0.01	0.76 ± 0.01
$\hbox {d}_{{SiGe}}$ (nm)	101.58 ± 0.13	105.85 ± 0.14	106.87 ± 0.15
$\hbox {d}_{{ox}}$ (nm)	2.66 ± 0.02	3.57 ± 0.03	3.21 ± 0.03
Inhomogeneity (%)	0.26 ± 0.02	2.39 ± 0.05	2.28 ± 0.05
**RMSE**	2.46	3.13	3.00

In our case, the fit quality given by the RMSE value (described in section “Methods”) is significantly better using the CL parameterization (Fig. 8). These smaller RMSE values indicate that the CL model describes the optical properties of the samples more accurately, while the smaller confidence limit values of the parameters and the smaller cross-correlations also justify the better suitability of this model. Figure 9 shows, however, that *n* and the gap features determined by both methods are very similar.

**Figure 6:**
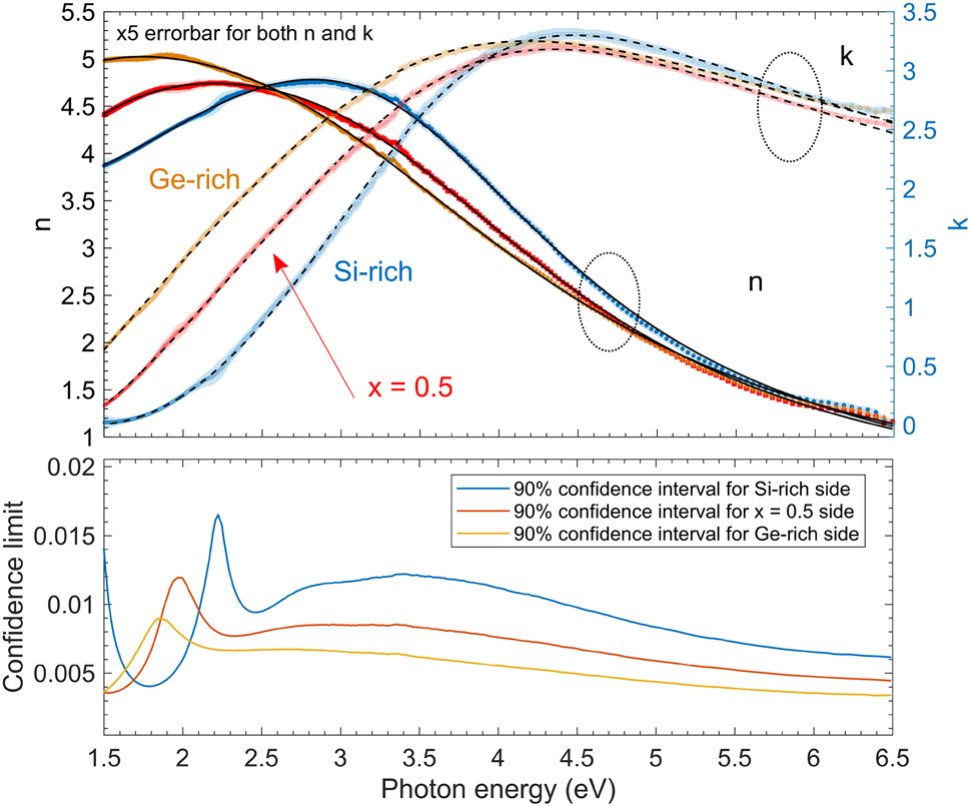
Optical properties of sample ‘C’ calculated by the CL model (dashed black lines) and by numerical inversion^53^ (colored lines with filled error bars) for three different locations on the sample. (The error bars for both *n* and *k* from the numerical inversion are magnified by a factor of 5 (Top figure) for a better visualization.)

For utilizing the full capacity of SE a numerical inversion study was also made^53^. Given the thickness values by the CL model, a model-free approach was also used to analyze the spectra further. This method can reveal hidden features in the spectra that could have been smoothed by the CL model. For this reason, the 3 measured ellipsometric $\Psi$-$\Delta$ pairs at the three angles of incidence (see section “Methods”) were fitted at each measurement wavelength to obtain (*n*,*k*) pairs independently. The result of this analysis is presented in Fig. 6. From the excellent agreement between the (*n*,*k*) pairs determined by the CL model and by the direct inversion we conclude that the CL model is appropriate for describing the sample in this photon energy range. The 90% confidence limit for both *n* and *k* were calculated using the results for numerical inversion.

In Fig. 9 all the fitted parameters of the CL oscillator are presented as a function of position *x*. The parameter variations were fitted using quadratic equations in the form of $y=p_1\cdot x^2+p_2\cdot x+p_3$, where $(p_1,p_2,p_3)$ are the fitted parameters, presented in Fig. 9 and also listed in Table 4 for each sample. Most importantly, for $E_g$ and (*n*, *k*), there is a monotonous increase and decrease with increasing concentration of H, respectively. For (*n*, *k*), linear equations were fitted, with a fixed value of $p_1 = 0$. The CL and TL models gave a similar result, despite the fact that $\varepsilon _1(\infty )$ has frequently a negative value in case of the TL model having nonphysical behaviour. This was observed mainly in positions with Ge-rich compositions.

The gap values are lower than that obtained by PECVD^54^. The $E_g$ values shown in Fig. 3 of Ref.^54^ decrease from approx. 1.6 to 1.2 eV as *x* increases from 0.0 to 0.4, whereas in our case $E_g$ spans a smaller range—from approx. 1.3 to 1.1 in the same range of *x*. Fitting the dependence of the parameters on *x* by a polynomial a good qualitative agreement can be found between our work and that from Ref.^54^, as revealed in Table 3. The lower gap values can be explained by the fact that magnetron sputtering is carried out using a mixture of H and Ar gases. The partial pressure of H was controlled by adding Ar to keep the total pressure at the value of $3 \times 10\,^{-3}$ mbar. Using these techniques it cannot be avoided that some Ar is incorporated in the layer as revealed in our earlier work^55^. Many other studies have dealt with the influence of the deposition condition of different techniques for the physical properties of amorphous thin films with similar conclusions in terms of the shifts in *n* and the band gap energy^56^ (Table 4).

**Table 3:** Fitted composition-dependence of the CL parameters (Eq. 3) in the position range of 0–8 mm (corresponding to $x=0.0 \ldots 0.4$) for each sample.

Sample	$p_H/p = 0$	$p_H/p = 0.05$	$p_H/p = 0.1$	$p_H/p = 0.2$	From Ref.^54^
Parameter	$\mathbf {(p_1,p_2,p_3)}$ parameters for $\mathbf {y=p_1\cdot x^2+p_2\cdot x+p_3}$, $\hbox {R}^2$				
A (eV)	(14.03,-37.7, 117.46), 0.97	(6.73, -11.69, 95.58), 0.98	(30.26, -44.89, 103.50), 0.97	(27.13, -46.89, 107.86), 0.99	(18.4, -42.5, 92.9), 0.74
$\Gamma$ (eV)	(− 0.02, 0.53, 2.70), 0.99	(0.03, 0.40, 2.94), 0.99	(− 0.04, 0.52, 3.02), 0.99	(− 0.25, 0.92, 2.86), 0.99	(− 2.33, 2.26, 2.54), 0.97
$\hbox {E}_0$ (eV)	(− 0.12, 0.33, 3.68), 0.99	(− 0.03, 9, 52*, 3.72), 0.84	(− 0.22, 0.30, 3.73), 0.96	(− 0.24, 0.41, 3.63), 0.98	−
$\hbox {E}_g^{CL}$ (eV)	(0.06, − 0.14, 0.944), 0.85	(0.05, − 0.21, 1.15), 0.98	(− 0.03, − 0.15, 1.33), 0.99	(− 0.06, − 0.16, 1.43), 0.99	(0.08, − 1.09, 1.70), 0.99
$\hbox {E}_p$ (eV)	(0.22, − 1.16, 1.99), 0.98	(0.13, − 0.49, 1.33), 0.99	(0.70, − 1.35, 1.61), 0.99	(0.63, − 1.39, 1.81), 0.99	(0.33, − 2.01, 1.35), 0.96

Results from Ref.^54^ are also presented. The values marked by ‘*’ are multiplied by $10^3$.

**Table 4:** Quadratic fit on the composition-dependence of the CL parameters (Eq. 3) in the whole position range of 20 mm for each sample.

Sample	$p_H/p = 0$	$p_H/p = 0.05$	$p_H/p = 0.1$	$p_H/p = 0.2$
Parameter	$\mathbf {(p_1,p_2,p_3)}$ parameters for $\mathbf {y=p_1\cdot x^2+p_2\cdot x+p_3}$, $\hbox {R}^2$			
$\varepsilon _1(\infty )$	(− 0.53*, 1.83*, 0.43), 0.87	(− 0.17*, − 0.99*, 0.88), 0.89	(− 0.57*, 2.17*, 0.70), 0.89	(− 1.19*, 0.25*, 0.68), 0.99
A (eV)	(0.16, − 3.76, 115.75), 0.94	(0.04, − 0.77, 94.56), 0.76	(0.11, − 2.17, 98.58), 0.68	(0.16, − 3.25, 103.85), 0.85
$\Gamma$ (eV)	(0.09*, 0.067, 2.70), 0.99	(− 0.32*, 0.06, 2.93), 0.99	(− 1.27*, 0.08, 2.99), 0.99	(− 1.35*, 9.62*, 2.87), 0.99
$\hbox {E}_0$ (eV)	(− 2.76*, 0.05, 3.67), 0.99	(− 0.64*, 0.99*, 3.73), 0.98	(1.67*, 0.02, 3.75), 0.908	(− 1.71*, 0.03, 3.65), 0.81
$\hbox {E}_g^{CL}$ (eV)	(− 0.30*, − 5.98*, 0.93), 0.99	(0.17*, − 0.02, 1.15), 0.99	(− 0.23*, − 0.02, 1.34), 0.99	(− 0.32*, − 0.02, 1.43), 0.99
$\hbox {E}_p$ (eV)	(4.39*, − 0.15, 1.99), 0.99	(0.91*, − 0.05, 1.31), 0.99	(2.80*, − 0.09, 1.50), 0.96	(3.51*, − 0.11, 1.73), 0.98
$\hbox {n}_{{CL}}$ @ 1.96 eV	(${\mathbf {0}}$, 0.02, 4.69), 0.99	(${\mathbf {0}}$, 0.02, 4.69), 0.985	(${\mathbf {0}}$, 0.03, 4.43), 0.98	(${\mathbf {0}}$, 0.04, 4.19), 0.99
$\hbox {k}_{{CL}}$ @ 1.96 eV	(${\mathbf {0}}$, 0.05, 0.35), 0.98	(${\mathbf {0}}$, 0.05, 0.42), 0.99	(${\mathbf {0}}$, 0.06, 0.21), 0.99	(${\mathbf {0}}$, 0.06, 0.11), 0.99
$\hbox {E}_g^TL$ (eV)	(0.52*, − 0.03, 1.15), 0.99	(0.01*, − 0.02, 1.19), 0.99	(0.03*, − 0.03, 1.39), 0.99	(0.43*, − 0.04, 1.49), 0.99
$\hbox {n}_{{TL}}$ @ 1.96 eV	(${\mathbf {0}}$, 0.02, 4.68), 0.99	(${\mathbf {0}}$, 0.04, 4.37), 0.99	(${\mathbf {0}}$, 0.03, 4.40), 0.99	(${\mathbf {0}}$, 0.05, 4.17), 0.99
$\hbox {k}_{{TL}}$ @ 1.96 eV	(${\mathbf {0}}$, 0.04, 0.36), 0.97	(${\mathbf {0}}$, 0.05, 0.41), 0.99	(${\mathbf {0}}$, 0.05, 0.22), 0.99	(${\mathbf {0}}$, 0.05, 0.14), 0.99

The values marked by ‘*’ are multiplied by 10$^3$. The $\hbox {R}^2$ values are also listed for each fit.

**Figure 7:**
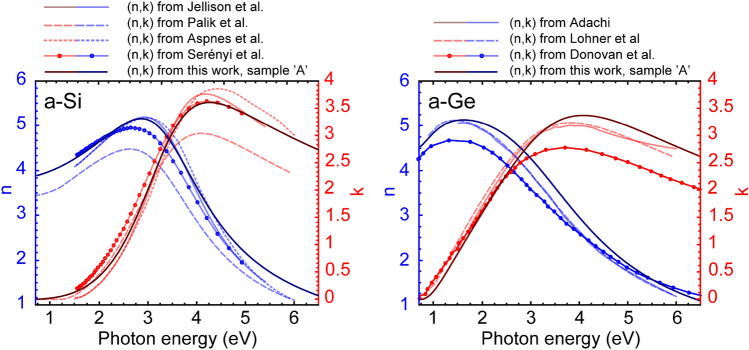
Comparison of the complex refractive index of a-Si (left-hand side) and a-Ge (right-hand side). The data are from Jellison et al.^57^, Palik et al.^18^, Aspnes et al.^58^, Serényi et al.^59^, Adachi^60^, Lohner et al.[submitted] and Donovant et al.^61^.

In case of PECVD a layer with higher density can be obtained, however, that technique is not capable of combinatorial sample preparation. It is also important to note that in this work the main goal was not to reproduce the literature values, but rather to investigate the main trends and dependencies as a function of composition and photon energy, the opportunities of parameterization and the description using simple formulas. These rules, and dependencies are basically independent of the shift caused by the Ar that was built in the layer during deposition. Furthermore, the optical function also shows a large variety depending on the deposition methods of both a-Si and a-Ge (see Fig. 7).

### Concentration dependence of the dielectric function

The amorphous $\hbox {Si}_{1-x}\,\hbox {Ge}_{{x}}$ alloy is an intrinsic semiconductor sputtered from undoped targets at relatively low temperature in our experiment. In a-Si and a-Ge the atoms have four electrons shared in covalent bonds with other adjacent atoms. The atoms are tetrahedrally coordinated, but there is no long-range order in the structure. In addition, the amorphous structure typically contains “dangling bond” sites in which Si and Ge atoms have only three nearest neighbors. Dangling bonds in amorphous semiconductors have orbital energies in the middle of the gap, and electrons in these states are effectively non-bonding. Because these dangling bond sites are relatively far away from each other, there is little orbital overlap between them, and electrons in these mid-gap states are therefore localized (Anderson localization)^62^. Amorphous $\hbox {Si}_{1-x}\,\hbox {Ge}_{{x}}$ is insulating because electrons near the center of the gap are not mobile in the lattice. These localized states create a mobility gap, which separates the localized states from their extended equivalents^63^. The concept of “crystalline” bandgap can be replaced by the idea of a mobility gap. The transmission profile has no abrupt edge to the absorption at the bandgap energy, rather, the optical absorption spreads and extends well into the gap region. However, by hydrogenating the dangling bonds will be passivated. This generates orbitals, the energies of which are outside of the mobility gap. Hydrogenation thereby lowers the density of states in the mobility gap, consequently the optical absorption related to $\varepsilon _2$ decreases monotonically depending on the incorporated H amount (the quantity of dangling bond passivation)^64^. A semiclassical analysis of amorphous Si:H is discussed in Ref.^65^. Referring to the general nature of this analysis we can conclude that a similar mechanism plays a role in shaping of the dielectric function of $\hbox {Si}_{1-x}\,\hbox {Ge}_{{x}}$ alloys.

In a-Si, the mobility or Tauc gaps lie mainly in between the direct ($\approx$ 3 eV) and indirect ($\approx$ 1.1 eV) gaps of c-Si. The location of the mobility/Tauc gap depends on the amount of disorder (mainly bond angle distortions). Fried et al. showed that ion implantation-amorphized (non-hydrogenated) Si has a gap value as low as 0.85 eV, mainly due to the higher amount of disorder and dangling bonds^66^. Our pure Si value lies between this value and that of c-Si (Fig. 9), due to the large amount of damage and dangling bonds (but less than in the case of direct ion implantation) caused by the plasma deposition not compensated by H (in the non-hydrogenated case). In Refs.^66^ and^67^ it was also pointed out that the dielectric function of a-Si and a-Ge substantially depends on the applied deposition method, e.g., chemical vapor deposition, sputtering or amorphization by ion implantation (a smaller optical band gap and a larger broadening in case of the ion implantation-amorphized Si^68^). The distribution of electron states around the gap energy may largely differ depending on the preparation conditions. Consequently, the fitted numerical gap value also depends on the applied distribution function^68^—in our case the constant dipole or momentum matrix approach, in Ref.^66^ the Davis-Mott plot, assuming that the densities of electron energy states in the valence and conduction bands near the band gap have a power function distribution, and the matrix elements for interband transition associated with photon absorption are equal for all transitions. In hydrogenated amorphous Si (a-Si:H) the mobility/Tauc gap is higher than in a-Si because roughly 10% of the chemical bonds in the amorphous 3D network are Si–H bonds ($\approx$ 3 eV) and because these are stronger than the Si–Si bonds ($\approx$ 2 eV).

While in the case of c-$\hbox {Si}_{1-x}\,\hbox {Ge}_{{x}}$ there are many features and parameters of the dielectric function (the composition dependence of which can also be parameterized)^43^, a-$\hbox {Si}_{1-x}\,\hbox {Ge}_{{x}}$ has mainly been characterized by the absorption onset, the electronic gap determined from the absorption features and the Tauc plot^42^. However, since the absorption of amorphous materials can accurately be described using a few parameters, the dielectric function can also be analyzed in the absorption region. Although the near-gap and below gap *n* values are more interesting from the application point of view, we reveal that the high photon energy region also shows systematic features allowing potential optical applications in the small wavelength region.

**Figure 8:**
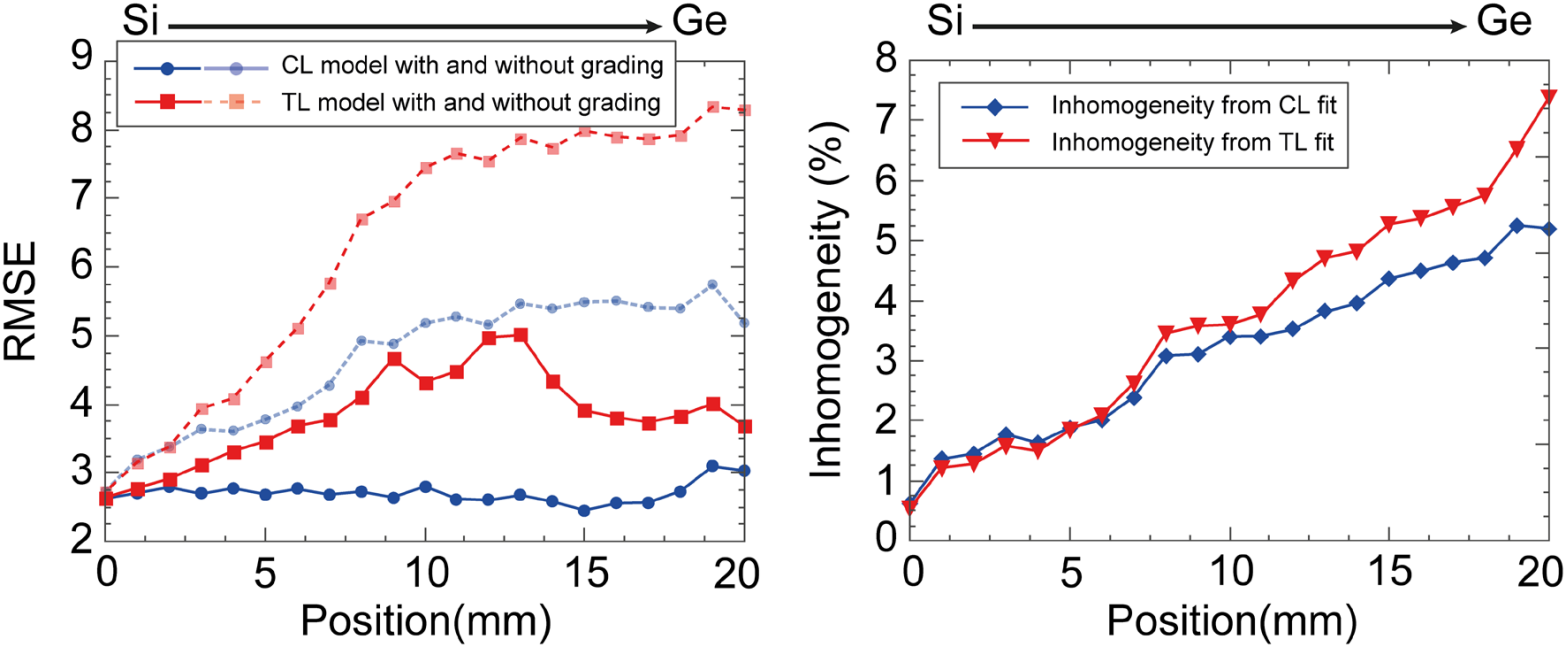
Quality of fit (RMSE—the smaller the better—see section “Methods”) along sample ‘D’ ($\hbox {p}_H$/p$=0.2$). The RMSE values related to the CL and TL models are shown using blue and red dotted lines, respectively. For both models, the best fit was achieved by using a graded layer, resulting in low values of inhomogeneity inside the a-$\hbox {Si}_{1-x}\,\hbox {Ge}_{{x}}$ layer. On the right-hand side, the percentage of the inhomogeneity is presented for both models.

In terms of the Si and Ge concentration ratio, besides reports dealing with the concentration dependent properties of c-$\hbox {Si}_{1-x}\,\hbox {Ge}_{{x}}$^25,30,43,69^, there are also numerous studies that focus on amorphous compounds^40,50,66–74^. Comprehensive parameterizations for both the photon energies and compositions were presented for crystalline materials, in many cases supported by density of states calculations^75^, also pointing out the Vegard’s law-like behavior^69^. In this study we further strictly focus on the amorphous cases. Most results were restricted to a limited range of wavelengths and compositions, therefore, a comprehensive basis for comparison is lacking. There was a significant amount of scattering in the obtained data, due to the large dependence of the properties on the preparation parameters. Fedala et al. measured optical gap values from 1.3 to 2.1 eV using absorption spectroscopy for compositions up to $x=0.6$, and an unknown crystallinity and partial pressure of H during magnetron sputtering^40^. Shahahmadi et al. found band gap values of $\approx$1.2 eV for both the amorphous and crystalline $\hbox {Si}_{1-x}\,\hbox {Ge}_{{x}}$ (created by annealing at temperatures up to 550 $^\circ$C) for a single composition of $x=0.77$^9^. Hernández-Montero et al. measured optical band gap values on a-$\hbox {Si}_{1-x}\,\hbox {Ge}_{{x}}$ prepared by low pressure chemical vapor deposition ranging from $\approx$0.8 to almost 2 eV, depending on the composition and on the method of evaluation^70^. They found *n* variations from $\approx$ 3.3 (Si) to $\approx$ 4.5 (Ge), in good agreement with our data for the $p_H/p=0.2$ case. In Ref.^70^, the $\hbox {H}_2$ flow was 1000 sccm, whereas that of the $\hbox {SiH}_4$ and $\hbox {GeH}_4$ were varied from 0 to 100 and 0 to 500 sccm, respectively. The thickness of the layers was varied from 680 to 1048 nm depending on the composition. In Ref.^50^, both the constant dipole matrix and the constant momentum matrix approach were compared and applied to a-$\hbox {Si}_{1-x}\,\hbox {Ge}_{{x}}$, resulting in gap values between 1.42 and 1.63 eV, the composition and H concentration of which was not exactly known. Perez et al. compared PECVD a-$\hbox {Si}_{1-x}\,\hbox {Ge}_{{x}}$ films for $p_H/p=0.05$, $x=0.0$, 0.1, 0.2, 0.5 and 1.0 from $\hbox {SiH}_4$+$\hbox {GeF}_4$ and from $\hbox {SiH}_4$+$\hbox {GeH}_4$ finding optical gap values that were not linear with the composition, and higher for the $\hbox {SiH}_4$+$\hbox {GeF}_4$ case, having values between $\approx$ 1.1 and $\approx$ 1.9 eV^71^. A co-sputtering (combinatorial) method using an r.f. system with a H/Ar ratio of 1.4/12 was demonstrated by Weisz et al.^72^ on a-$\hbox {Si}_{1-x}\,\hbox {Ge}_{{x}}$ films for $x=0.30$, 0.50, 0.61, 0.77 and 0.93 resulting in optical gap values between 1.1 and 1.7 eV using the Cody^47^ fit. Better photoconductance was found at the Si-rich side explained by the H that preferentially bonds to Si. Magnetron co-sputtering was also used by Dimova-Malinoska et al.^73^ to create a-$\hbox {Si}_{1-x}\,\hbox {Ge}_{{x}}$ films with Ge concentrations from $x=0.06$ to 0.38 at $p_H/p=0.05$, and corresponding optical band gaps from $\approx$ 1.1 to $\approx$ 1.8 eV. R.f. glow discharge a-$\hbox {Si}_{1-x}\,\hbox {Ge}_{{x}}$ films deposited at high H flow ratios were compared for anodic and cathodic depositions by Wickboldt et al.^74^ finding optical gap values between 1.1 and 1.9 eV, being higher for the cathodic case (Fig. 8).

**Figure 9:**
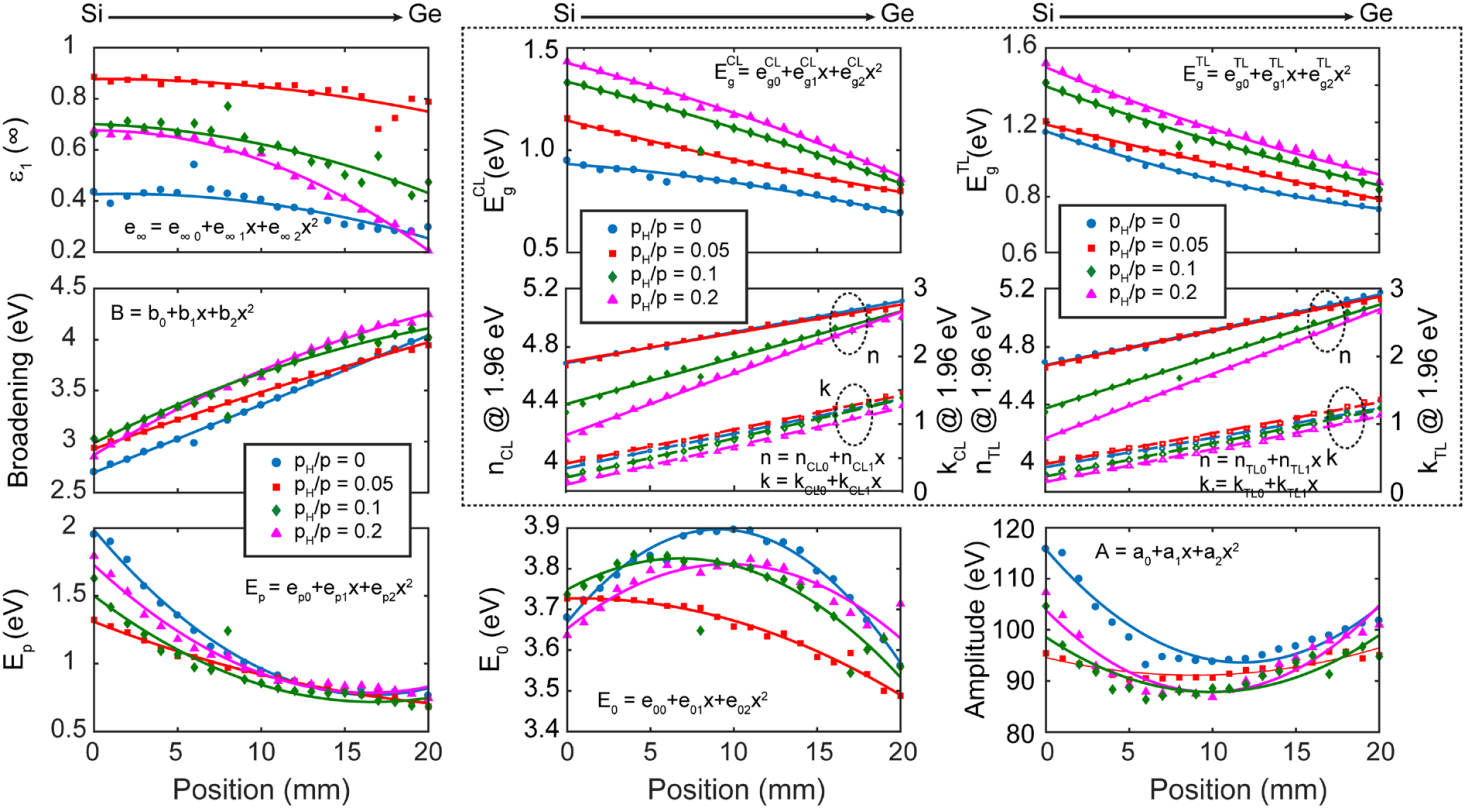
Optical gaps of a-$\hbox {Si}_{1-x}\,\hbox {Ge}_{{x}}$ samples and the real and imaginary parts of the complex refractive indices at the wavelength of 633 nm (photon energy of 1.96 eV) modeled by CL (left-hand side) and TL (right-hand side) oscillators (graphs in the dotted frame). The other graphs show the *x* dependence of the fitted parameters utilizing the CL dispersion of Eq. (3), with the Broadening and Amplitude denoted by $\Gamma$ and *A*, respectively.

**Figure 10:**
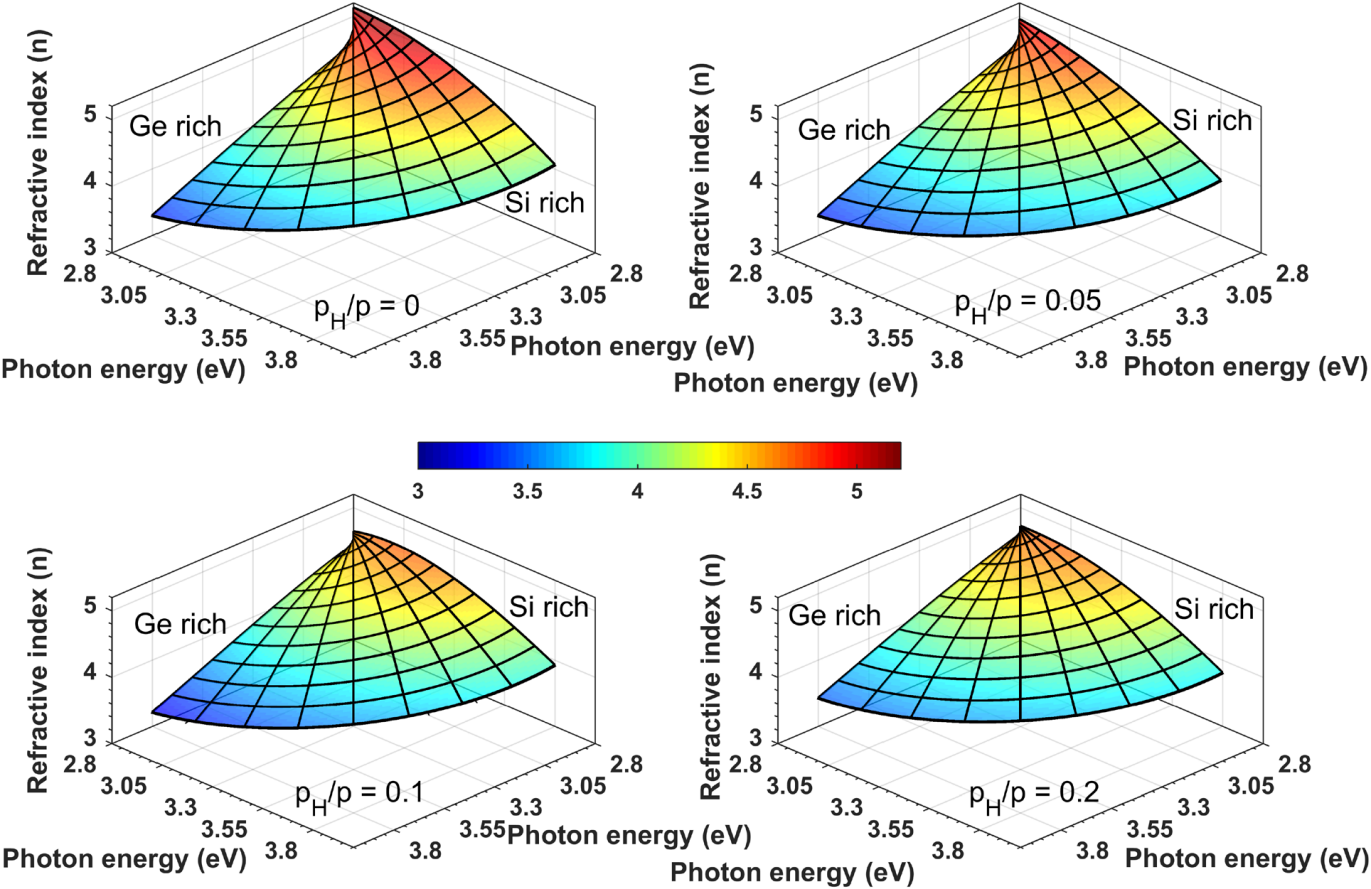
Vegard plots in the linear range of *n* for all the samples of different $\hbox {p}_H/p$ values.

**Figure 11:**
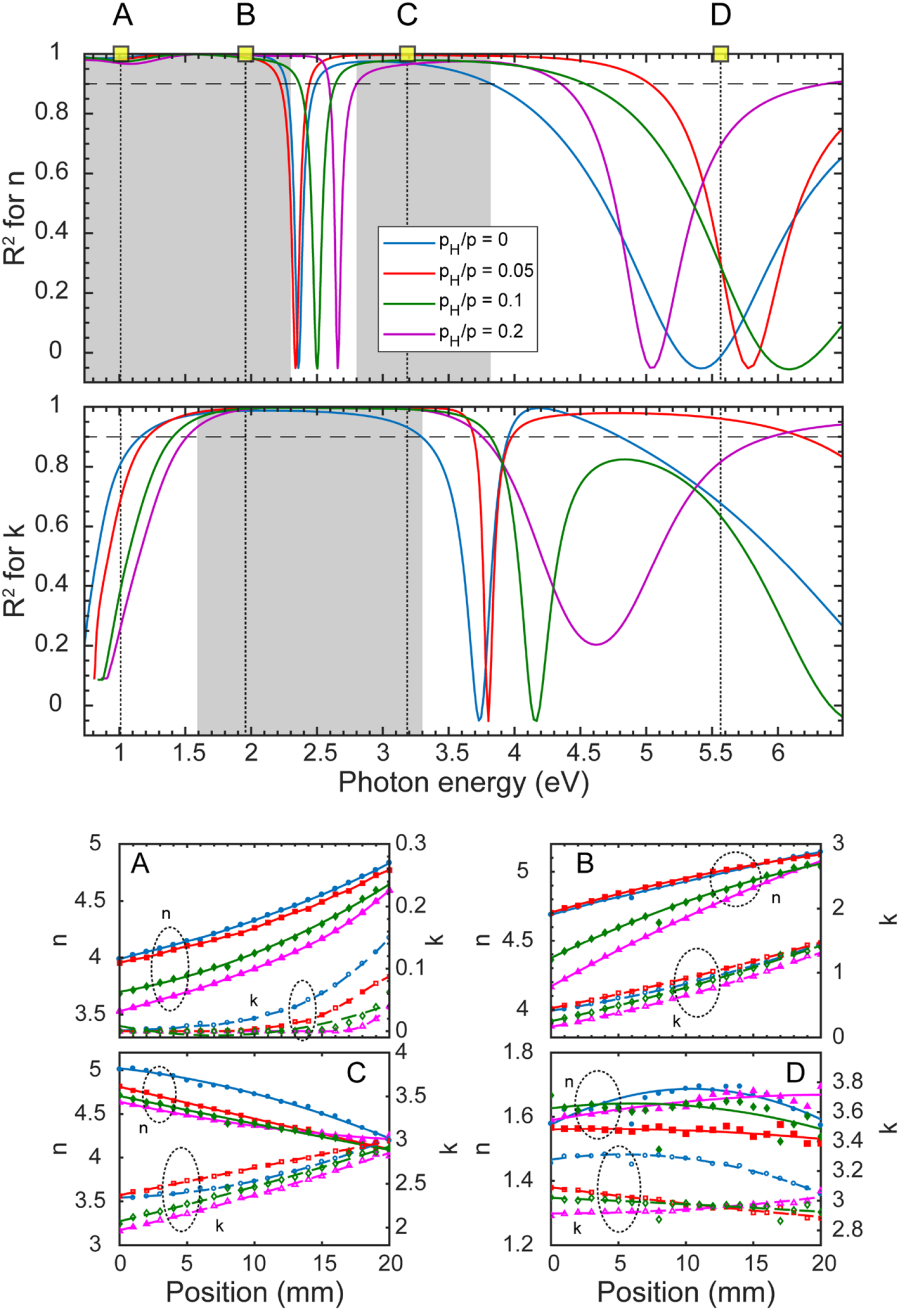
Dispersion of the linearity (*R*^2^—defined in Eq. 5) of the complex refractive index (both *n* and *k* spectra) with the concentration. The curves of different colors on the top graph correspond to different $p_H/p$ values. The highlighted photon energies are the following: $A=1.01$ eV, $B=1.96$ eV, $C=3.14$ eV and $D=5.56$ eV. The grey areas show the photon energy range of linear behavior of the real and imaginary parts of the complex refractive index for each sample.

Figure 9 shows *n* and *k* values for $p_{H}/p=0.0$ to 0.2 at the photon energy of 1.96 eV (the He–Ne laser wavelength of 633 nm). The CL and the TL models result in almost identical *n* and *k* values, which are close to the values found in previous studies discussed above. The linearity regarding both the photon energy and the composition is analyzed using a Vegard plot and numerical correlation values in Figs. 10 and 11. Figure 9 shows qualitatively that at this photon energy the variation of the optical properties is linear with the composition with lower values of both *n* and *k* for higher concentration of H ($p_H/p$). The Vegard-like behavior of *n* is presented by a polar coordinate system in Fig. 10, showing the linear dependence on the composition in the photon energy range from 2.8 to 4.5 eV in the case of Sample ‘C’—with the linearity further analyzed numerically in Fig. 9. The two perpendicular planes at the back of the plot show the wavelength dependence of *n* for $x=0$ and 1, and the conical surface illustrates the change of *n* with composition *x*. The optical gap values of the CL and TL models are very similar for lateral positions above 10 mm (higher *x* values), however, there is a significant deviation in the Si-rich positions. According to Fig. 8, the fit quality is much better in case of the CL model for most of the compositions, especially for the Ge-rich side. In both cases, the gap values increase with increasing concentration of H, due to the higher Si–H bond energy (see above).

The linearity of both *n* and *k* are shown in the whole investigated photon energy range in Fig. 11. In order to explore the photon energy range where the optical properties of the a-$\hbox {Si}_{1-x}\,\hbox {Ge}_x$:H samples change proportional to the composition *x*, a linear function was fitted at each separate wavelength for all *x* values. Subsequently, the quality of these fits were described by the adjusted $R^2$ values of the fits defined by:

5 $$\begin{aligned} R^2=1-\frac{N-1}{N-P}\cdot \frac{SSE}{SST}, \end{aligned}$$

where *SSE* is the sum of squared error, *SST* is the sum of squared total, *N* is the number of observations, and *P* is the number of regression coefficients. In Fig. 11, $R^2$ is plotted for both *n* and *k* for each sample. The linearity of the optical properties with composition is excellent for all samples in the ranges, indicated by gray shadings ($R^2 = 0.9$ is indicated by vertical dotted lines for each sample). Comparing this results with the features shown in the left-hand side graph of Fig. 5, the regions of best linearity in Fig. 11 correspond to ranges of Fig. 5 in which the values change significantly, because of the shifted peaks in both *n* and *k*. The linearity deteriorates close to the peaks of both *n* and *k*, while it is best at the steep parts. These features, the positions of which can be identified also in Fig. 5, are shifted due to the changing concentration of H. In the bottom part of Fig. 11 four photon energies were selected to illustrate the behaviour of *n* and *k*. One remarkable feature to point out is the linear dependence of the optical properties on both position and composition in the photon energy ranges identified in Fig. 11. Consequently, the composition dependence of the optical properties can be approximated using equations

6 $$\begin{aligned} n_{Si_{1-x}Ge_{x}}(x)= (1-x)\cdot n_{Si}+ x\cdot n_{Ge} \end{aligned}$$

and

7 $$\begin{aligned} k_{Si_{1-x}Ge_{x}}(x)= (1-x)\cdot k_{Si}+ x\cdot k_{Ge} \end{aligned}$$

in broad ranges of wavelengths (see Fig. 11). Using this relation $(n_{Si},n_{Ge})$ and $(k_{Si},k_{Ge})$ can be defined for photon energies in the linear ranges of Fig. 11. In Table 5, the Vegard coefficients are presented for the photon energies of 1.96 eV and 3.14 eV.

**Table 5:** Parameters $(n_{Si},n_{Ge})$ and $(k_{Si},k_{Ge})$ of the Vegard-like behavior at the photon energies of 1.96 eV and at 3.14 eV based on Eqs. (6) and (7).

Sample	$p_H/p = 0$	$p_H/p = 0.05$	$p_H/p = 0.1$	$p_H/p = 0.2$
$(n_{Si},n_{Ge})$ @ 1.96eV	(4.93, 5.15)	(4.94, 5.15)	(4.75, 5.10)	(4.66, 5.10)
$(k_{Si},k_{Ge})$ @ 1.96eV	(0.87, 1.38)	(0.93, 1.44)	(0.79, 1.38)	(0.68, 1.26)
$(n_{Si},n_{Ge})$ @ 3.14eV	(4.69, 4.29)	(4.45, 4.09)	(4.39, 4.08)	(4.39, 4.17)
$(k_{Si},k_{Ge})$ @ 3.14 eV	(2.59, 2.92)	(2.68, 2.99)	(2.48, 2.91)	(2.39, 2.82)

### Concentration dependence of H incorporation

In general, the deposition rate will be given by a function that depends on the substrate temperature, source-to-substrate distance and power, as well as effects related to the sputtering pressure and sputtering gas composition. In all measurements reported here, the substrate temperature, sputtering pressure of the gas mixture and source-to-substrate distance are held constant. The rates for Si and Ge are

8 $$\begin{aligned} R_{Si}&= {} A\cdot f(P_{Si})\cdot f(p_H), \end{aligned}$$

9 $$\begin{aligned} R_{Ge}&= {} B\cdot g(P_{Ge})\cdot g(p_H), \end{aligned}$$

where $f(P_{Si})$, $g(P_{Ge})$ represent the power dependence of the rate, whereas $f(p_H)$ and $g(p_H)$ denote the effect due to added H for Si and Ge, respectively. These terms are not mutually independent. However, they can be separated on the basis of the different physical mechanisms for rate modification.

During the deposition process the power of both magnetron targets was chosen to keep the deposition rate at the same value of 0.4 nm/s separately. The power of the two magnetron sources is regulated synchronously with the position according to the anticipated *x* composition:

10 $$\begin{aligned} f(P_{Si})&=x\cdot P^0_{Si}, \end{aligned}$$

11 $$\begin{aligned} g(P_{Ge})&=(1-x)\cdot P^0_{Ge}. \end{aligned}$$

Investigating the effect of added H we find that the deposition rate decreases with increasing partial pressure of H. This reduces the effective sputtering rate when the H is present with relatively low concentrations in the DC sputtering:

12 $$\begin{aligned} {\frac{f(p_H)}{f(p_H=0)}} < 1, \end{aligned}$$

13 $$\begin{aligned} {\frac{g(p_H)}{g(p_H=0)}} < 1. \end{aligned}$$

The insensitivity to low concentrations of H in the magnetron case, although keeping the effective sputtering rates high, may reduce the incorporation of H into the a-Si and a-Ge matrix. Hydrogenation may occur through ionization of molecular H followed by reactions in the vapor phase or through reactions at the substrate^76^. Furthermore, the reactions between elemental target atoms and the reactive gas will change a fraction of the target’s atoms to compound molecules^77^. If hydrogenation occurs primarily from the formation of H ions or through atomic H (a certain percentage of $\hbox {H}^+$ ions accelerated towards the cathode will be neutralized and backscattered to the substrate) then the low ionization rate for H in the magnetron system may limit the efficiency of hydrogenation of the a-Si and a-Ge. Whichever model is taken to be based, the common feature is that the amount of the incorporated H is proportional to the partial pressure of H and the plasma current. The current is proportional to the square root of the power applied on the different targets, and the incorporation can be characterized by the ratio of the atomic concentrations of $C_H(Si)$ for pure Si and $C_H(Ge)$ for pure Ge, hence

14 $$\begin{aligned} {\frac{C_H(Si)}{C_H(Ge)}}=\sqrt{\frac{P^0_{Si}}{P^0_{Ge}}} \approx 2, \end{aligned}$$

in agreement with the ERDA measurements of Fig. 3. It can be concluded that the amount of incorporated H is proportional to the plasma current which otherwise can be measured as the sum of the currents flowing through the targets.

## Methods

### Spectroscopic ellipsometry

Spectroscopic ellipsometry is an attractive tool for thin film characterization. This method is based on the measurement of both the amplitude- and phase change of the light, which is reflected on the surface of the sample. From the measurement the ellipsometric angles, $\Psi$ and $\Delta$ are usually presented and they are defined by

15 $$\begin{aligned} \rho =\tan \Psi \exp (\text {i}\Delta ), \end{aligned}$$

where $\rho$ is the complex reflectance ratio. The angles $\Psi$ an $\Delta$ are related to the amplitude ratio and the phase difference between p- and s-polarized light, respectively.

The 25 mm by 10 mm samples were scanned by a Woollam M-2000DI rotating compensator spectroscopic ellipsometer with a focused spot that were moved along the center line, parallel to the long edge of the sample. The measurements were carried out using a lateral resolution of 1 mm. The plane of incidence was parallel to the short edge, and the angle of incidence was varied between 60$^\circ$ and 70$^\circ$. The corresponding size of the focused spot was 0.3 mm wide and 0.6–0.9 mm long. The measurement time was a few seconds for one point and one angle of incidence in the whole wavelength range of 191–1690 nm (photon energies of 0.7–6.5 eV). As a result of the applied technique we obtained, within reasonable time, high-resolution and high-accuracy maps of optical properties as a function of composition, incident angle and wavelength.

**Figure 12:**
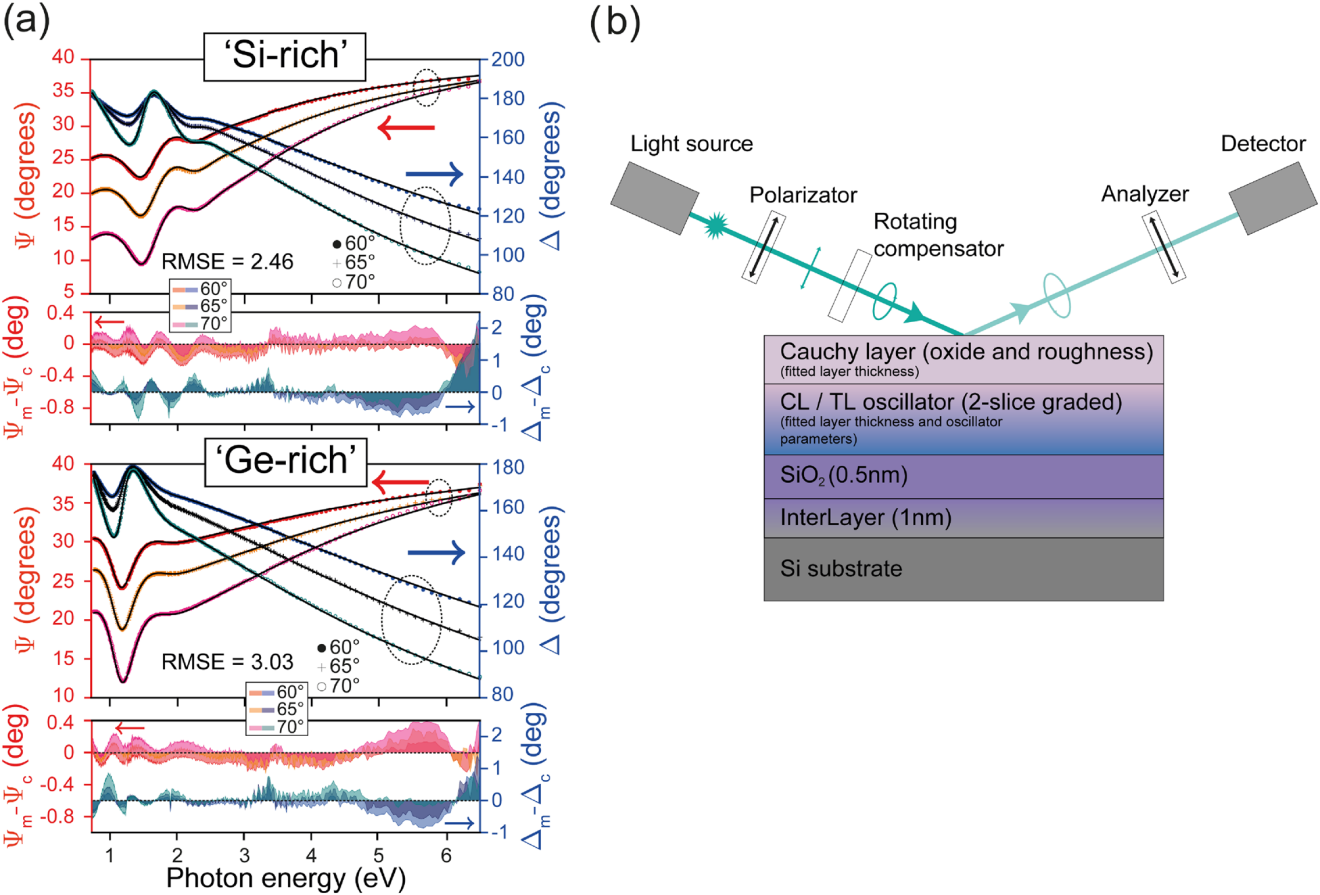
Ellipsometry spectra of the non-hydrogenated a-$\hbox {Si}_{1-x}\,\hbox {Ge}_{{x}}$ sample (‘A’) at the Si-rich and Ge-rich sides for different angles of incidence measured (symbols) and fitted (solid lines) by the CL dispersion. The difference between the measured ($\Psi _m, \Delta _m$) and calculated ($\Psi _c, \Delta _c$) ellipsometric angles is also included (**a**). The optical model and the schematic measurement configuration are shown on the right-hand side (**b**).

An optical model of multiple layers was constructed for an appropriate sample analysis (Fig. 12). This model consists of a bulk Si substrate, a thin $\hbox {SiO}_2$ layer with fixed thickness of 0.5 nm on the substrate and an interlayer (fixed at 1 nm) between the Si substrate and the $\hbox {SiO}_2$ layer. The optical properties of these materials are from Ref.^78^. The sputtered a-$\hbox {Si}_{1-x}\,\hbox {Ge}_{{x}}$ layer was described by a single oscillator model.

During the data evaluation the sensitive oscillator parameters and the thicknesses of the top two layers were fitted. The fitted values were calculated by minimizing the root mean square error (RMSE) defined by

16 $$\begin{aligned} \text {RMSE}=\sqrt{\frac{1}{3n-m}\sum \limits _{j=1}^{n}\left[ \left( \frac{N_{j}^{m}-N_{j}^{c}}{\sigma _{N_{j}}^{m}}\right) ^{2}+\left( \frac{C_{j}^{m}-C_{j}^{c}}{\sigma _{C{j}}^{m}}\right) ^{2}+\left( \frac{S_{j}^{m}-S_{j}^{c}}{\sigma _{S{j}}^{m}}\right) ^{2} \right] }, \end{aligned}$$

where *n* and *m* are the number of wavelengths and fit parameters, respectively, while $N=\cos (2\Psi )$, $C=\sin (2\Psi )\cos (\Delta )$ and $S=\sin (2\Psi )\sin (\Delta )$. The subscripts ‘*m*’ and ‘*c*’ indicate the measured and calculated values, while $\sigma$ is the standard deviation of the measured values. A global fit on random grid with Levenberg-Marquardt algorithm was used for obtaining the global minimum during the fitting process.

### Rutherford backscattering spectrometry

1.6-MeV ERDA and RBS measurements were made in a scattering chamber with a two-axis goniometer, which was connected to the 5-MV EG-2R Van de Graaff accelerator of the Wigner FK RMI. The $^4\,\hbox {He}^+$ analyzing ion beam was collimated usign two sets of slits with four-sectors. The spot was 0.2 mm wide and 1 mm high. The beam divergence was kept below 0.06$^\circ$. A transmission Faraday cup was used to measure the beam current. The vacuum was $\approx 10^{-4}$ Pa in the scattering chamber. The hydrocarbon deposition was avoided by liquid $\hbox {N}_2$ cooled traps along the beam path and around the wall of the chamber. ORTEC Si surface barrier detectors were used to detect ERDA and RBS spectra mounted at scattering angles of $\Theta = 165^\circ$ (RBS) and $\Theta = 20^\circ$ (ERDA). The resolution of the detector was 20 keV for RBS and somewhat higher for ERDA. In the latter case, to capture the scattered $\hbox {He}^+$ ions and separate them from the H particles to be detected, a 6-$\upmu$m thick Mylar foil was placed in front of the detector. The spectra were measured using sample tilt angles of 7$^\circ$ and 60$^\circ$ for RBS and 80$^\circ$ for ERDA. The RBX code was used to simulate the spectra^39^.

**Figure 13:**
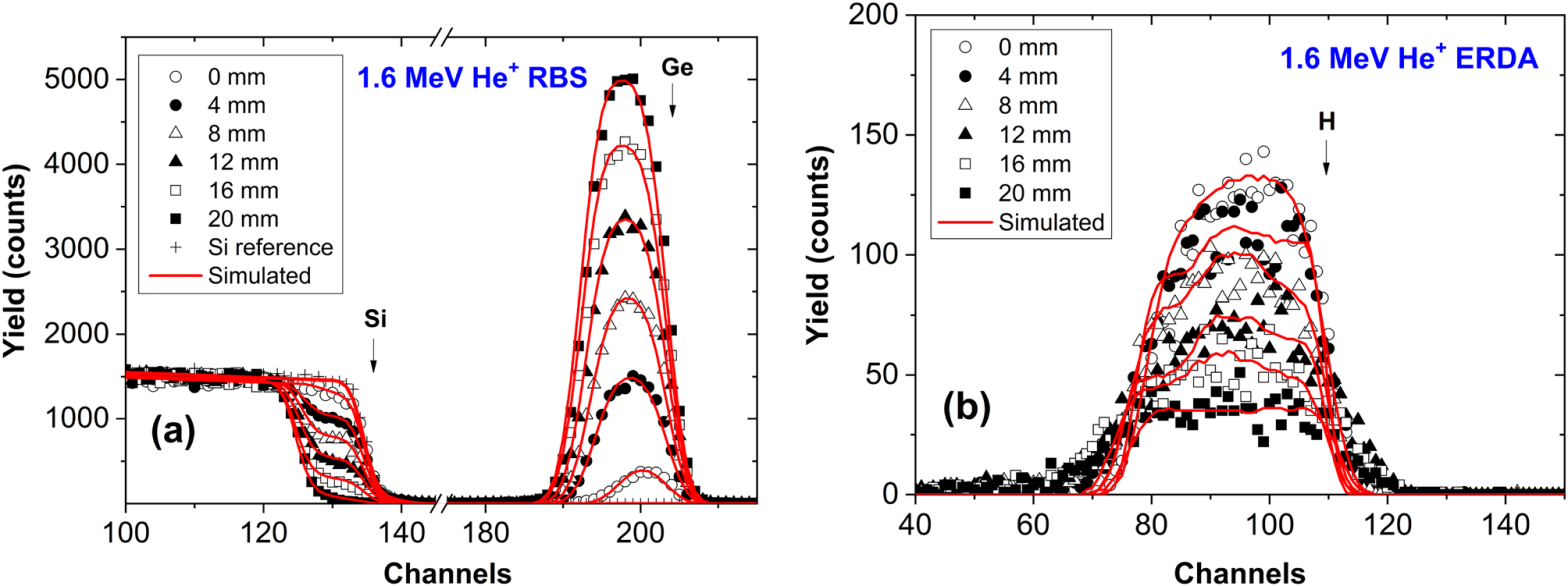
1.6 MeV $\hbox {He}^+$ (**a**) RBS and (**b**) ERDA spectra as a function of the lateral position along the sample, for an a-$\hbox {Si}_{1-x}\,\hbox {Ge}_{{x}}$:H layer with nominally 20% H content and with deposition rate of 0.4 nm/s. In (**a**), for comparison, a reference spectrum for a bare Si sample is also shown. Surface edges for Si, Ge, and H are represented by arrows.

Figure 13 shows 1.6 MeV $\hbox {He}^+$ RBS and ERDA spectra as a function of the lateral position along the center line of the sample parallel to the long edge, for an a-$\hbox {Si}_{1-x}\,\hbox {Ge}_{{x}}$:H layer with nominally 20% H content and with deposition rate of 0.4 nm/s. In Fig. 13a, for comparison, a reference spectrum for a bare Si sample is also shown. Surface edges for Si, Ge, and H are represented by arrows. As Fig. 13 shows, the decrease of the Si-yield between channels 125–140, and the increase of the Ge peak between channels 185–210 can be observed, as the sample position changes from 0 mm (Si-rich edge of the sample) to higher values. Meanwhile, the H peak in Fig. 13b decreases significantly when approaching the Ge-rich edge of the sample (20 mm). Red lines show the results of RBX simulations considering appropriate Si:Ge:H ratios in order to fit both the RBS and the ERDA spectra with the same model structure. Note that in general, almost fully homogeneous atomic concentrations were found in the a-$\hbox {Si}_{1-x}\,\hbox {Ge}_{{x}}$:H layers as a function of depth. The evaluated Si, Ge, and H contents, shown in Fig. 3, are averaged over the depth for the a-$\hbox {Si}_{1-x}\,\hbox {Ge}_{{x}}$:H layers.

### Atomic force microscopy

AFM measurements were performed using an instrument manufactured by AIST-NT (SmartSPM 1000). The instrument was used in tapping mode on a scanned area of 1 $\upmu$m by 1 $\upmu$m. The AFM images were evaluated applying several features of the Gwyddion software^79^ including data leveling, background subtraction and false color mapping.

**Figure 14:**
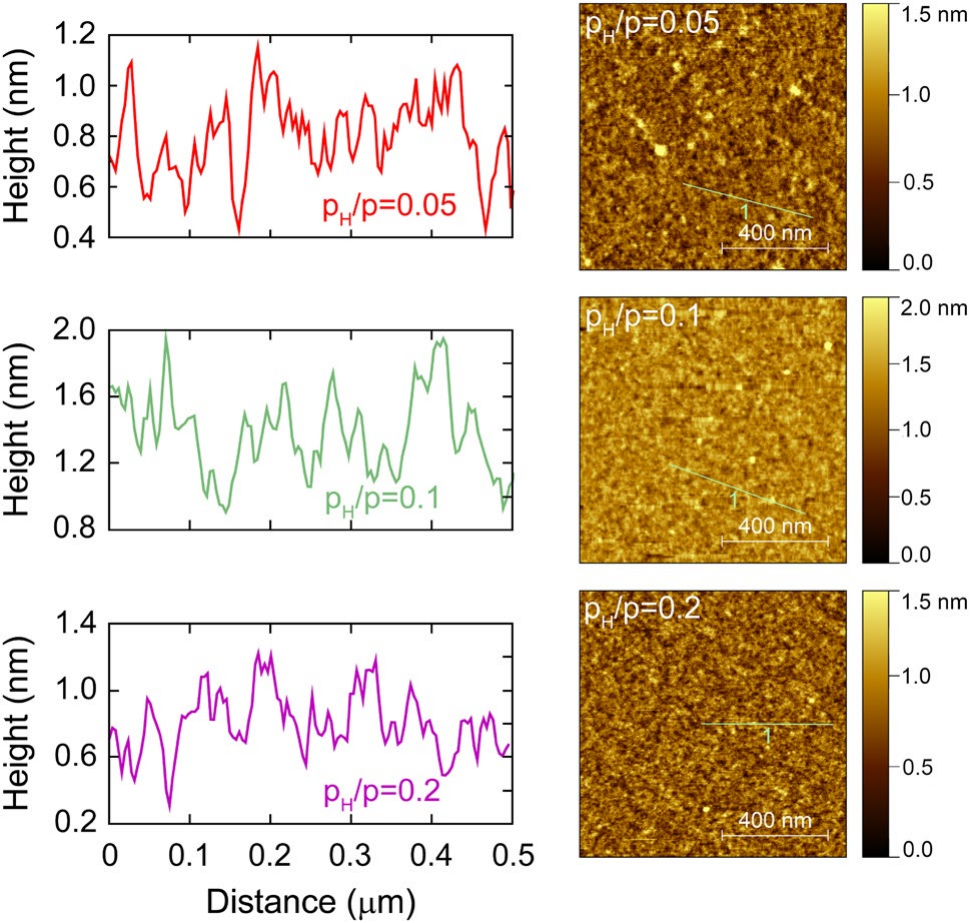
Line profiles and AFM topography images on the samples with $p_H/p=0.05$, 0.1 and 0.2.

Figure 14 shows surface topographies of the samples with $p_H/p$ values of 0.05, 0.10 and 0.20. The root mean square roughness values for all the three cases are around 0.2 nm. Based on these result, the surface roughness was not modelled for the ellipsometry evaluations as a separate layer. The minor effect of this surface roughness is included in the fitted thickness of the surface oxide layer. The overall good fit quality (see Fig. 12) shows the relevance of this approach and leads to reliable fitted dielectric function data.

## Supplementary information

Supplementary Information 1

Supplementary Information 2
