# Supplementary material for: Micro-combinatorial sampling of the optical properties of hydrogenated amorphous Si1-x Gex for the entire range of compositions towards a database for optoelectronics
Source: Sci Rep. 2020 Nov 6;10:19266. doi: 10.1038/s41598-020-74881-5 (PMC7648109; doi:10.1038/s41598-020-74881-5)
Supplement: Supplementary file 1 — Supplementary Information 1 [file 41598_2020_74881_MOESM1_ESM.pdf]

SUPPLEMENTARY INFORMATION

Micro-combinatorial sampling of the optical properties of hydrogenated amorphous $Si_{1-x}Ge_x$ for the entire range of compositions towards a database for optoelectronics

Benjamin Kalas[1,+], Zsolt Zolnai[1,+], György Sáfrán[1,+], Miklós Serényi[1,+], Emil Agócs[1,+], Tivadar Lohner[1,+], Attila Nemeth[2,+], Nguyen Quoc Khanh[1,+], Miklós Fried[1,3,+], and Peter Petrik[1,*,+]

[1]Institute for Technical Physics and Materials Science, Centre for Energy Research, Konkoly-Thege Rd. 29-33, 1121 Budapest, Hungary

[2]Institute for Particle and Nuclear Physics, Wigner Research Centre for Physics, Konkoly-Thege Rd. 29-33, 1121 Budapest, Hungary

[3]Institute of Microelectronics and Technology, Obuda University, Tavaszmezo u. 17, H-1084 Budapest, Hungary´

[*]petrik@mfa.kfki.hu

[+]these authors contributed equally to this work

Tabulated data

The refractive index (n) and extinction coefficient (k) spectra of $Si_{1-x}Ge_x$ determined using the best fit Cody-Lorentz parametric model is attached as a tabulated data file for compositions of x= 0..1, photon energies of E = 0.7..6.5 eV and partial H pressures of P_H/p= 0..0.2. Fig. 1 shows a rendered image of the tabulated data (Fig. 4 in the manuscript).

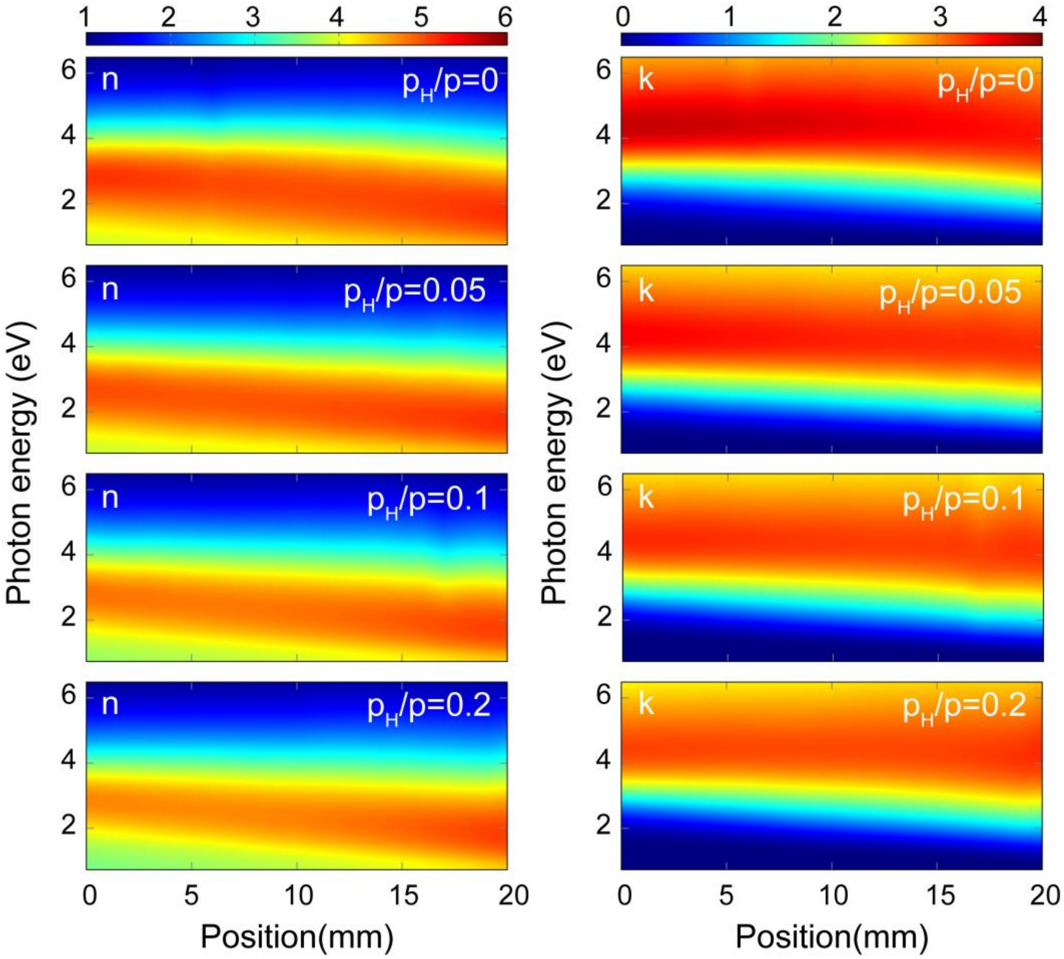

Figure 1. Real and imaginary parts of the complex refractive indices of a-$Si_{1-x}Ge_x$:H thin films (left and right column, respectively) with different partial pressures of H (p_H/p= 0,0.05, 0.1 and 0.2) as a function of both the lateral position along the 20 mm long gradient section and photon energy. In accordance with the RBS plots, the zero position corresponds to the Si-rich side of the sample.
